# Supplementary material for: Research on the path of green technology innovation driven by the Environmental Protection Tax Law: Based on data of heavy polluting enterprises
Source: PLoS One. 2024 Jul 30;19(7):e0308215. doi: 10.1371/journal.pone.0308215 (PMC11288438; doi:10.1371/journal.pone.0308215)
Supplement: S1 Data — (DOC) [file pone.0308215.s001.doc]

The symbols in the table represent the following elements:

**Main independent variable**

xt: Coordination between levies and administrations

jc: Emissions monitoring science

cy: Differential tax rate setting

gx: Tax information sharing

fw: Definition of the scope of levy and administration

fd: Tax declaration counseling

wz: Accountability mechanism design

yh: Implementation of preferential policies

sl: Tax rate level verification

**Controlled variable**

me: Medium-sized enterprise

be: Large-sized enterprise

pe: Private enterprise

fe: Foreign-funded enterprise

**Explained variable**

Gcra: Green process innovation

Gcom: Green product innovation

Gend: End treatment innovation

Gtec: Green technology innovation

|  | 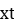 | 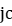 | 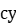 | 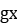 | 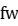 | 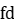 | 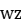 | 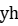 | 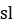 | 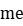 | 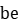 | 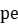 | 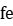 | 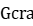 | 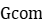 | 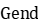 | 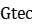 |
| --- | --- | --- | --- | --- | --- | --- | --- | --- | --- | --- | --- | --- | --- | --- | --- | --- | --- |
| S1 | 1 | 1 | 2 | 1 | 2 | 1 | 2 | 1 | 1 | 0 | 0 | 1 | 0 | 2 | 1 | 1 | 2 |
| 2 | 4 | 3 | 4 | 5 | 3 | 4 | 5 | 4 | 5 | 1 | 0 | 0 | 0 | 3 | 4 | 5 | 3 |
| 3 | 5 | 6 | 4 | 5 | 5 | 4 | 3 | 4 | 5 | 0 | 0 | 0 | 1 | 3 | 4 | 5 | 3 |
| 4 | 4 | 3 | 5 | 4 | 3 | 5 | 3 | 4 | 3 | 1 | 0 | 1 | 0 | 5 | 3 | 3 | 4 |
| 5 | 6 | 5 | 7 | 7 | 3 | 7 | 5 | 6 | 5 | 0 | 0 | 1 | 0 | 7 | 5 | 7 | 6 |
| 6 | 3 | 2 | 3 | 2 | 3 | 4 | 3 | 2 | 3 | 0 | 1 | 1 | 0 | 4 | 3 | 4 | 5 |
| 7 | 4 | 5 | 3 | 4 | 3 | 4 | 3 | 4 | 3 | 1 | 0 | 0 | 0 | 5 | 3 | 4 | 3 |
| 8 | 3 | 5 | 4 | 3 | 5 | 3 | 4 | 5 | 4 | 1 | 0 | 1 | 0 | 3 | 4 | 3 | 4 |
| 9 | 2 | 3 | 2 | 2 | 3 | 3 | 3 | 3 | 2 | 0 | 0 | 1 | 0 | 2 | 3 | 2 | 2 |
| 10 | 3 | 2 | 2 | 3 | 4 | 5 | 3 | 4 | 5 | 0 | 1 | 1 | 0 | 4 | 4 | 3 | 4 |
| 11 | 5 | 5 | 4 | 5 | 3 | 5 | 4 | 3 | 4 | 0 | 0 | 1 | 0 | 4 | 5 | 3 | 5 |
| 12 | 4 | 3 | 4 | 3 | 5 | 4 | 3 | 4 | 5 | 1 | 0 | 0 | 0 | 5 | 4 | 5 | 4 |
| 13 | 3 | 4 | 5 | 3 | 4 | 3 | 5 | 4 | 3 | 0 | 0 | 0 | 1 | 5 | 5 | 4 | 4 |
| 14 | 4 | 4 | 5 | 3 | 4 | 3 | 5 | 4 | 3 | 0 | 1 | 1 | 0 | 5 | 4 | 3 | 5 |
| 15 | 5 | 5 | 4 | 3 | 4 | 5 | 3 | 4 | 5 | 1 | 0 | 0 | 0 | 5 | 4 | 3 | 4 |
| 16 | 5 | 4 | 5 | 3 | 4 | 5 | 4 | 5 | 3 | 0 | 0 | 1 | 0 | 4 | 5 | 5 | 4 |
| 17 | 5 | 3 | 4 | 5 | 4 | 4 | 5 | 7 | 6 | 0 | 1 | 0 | 0 | 5 | 4 | 7 | 6 |
| 18 | 3 | 5 | 4 | 5 | 4 | 5 | 5 | 4 | 3 | 1 | 0 | 1 | 0 | 4 | 5 | 6 | 4 |
| 19 | 5 | 5 | 4 | 6 | 4 | 5 | 3 | 4 | 5 | 1 | 0 | 0 | 0 | 4 | 5 | 3 | 5 |
| 20 | 2 | 1 | 2 | 1 | 1 | 2 | 1 | 2 | 1 | 1 | 0 | 1 | 0 | 1 | 1 | 2 | 2 |
| 21 | 3 | 5 | 5 | 3 | 3 | 4 | 3 | 4 | 5 | 0 | 0 | 0 | 1 | 5 | 3 | 4 | 5 |
| 22 | 2 | 3 | 4 | 3 | 3 | 3 | 2 | 3 | 4 | 0 | 0 | 0 | 0 | 3 | 4 | 2 | 3 |
| 23 | 5 | 6 | 4 | 4 | 4 | 5 | 4 | 5 | 5 | 0 | 1 | 1 | 0 | 4 | 5 | 4 | 5 |
| 24 | 2 | 2 | 2 | 1 | 1 | 1 | 2 | 2 | 1 | 0 | 0 | 1 | 0 | 3 | 1 | 1 | 2 |
| 25 | 5 | 6 | 5 | 4 | 4 | 5 | 4 | 5 | 3 | 1 | 0 | 1 | 0 | 4 | 3 | 4 | 5 |
| 26 | 6 | 5 | 5 | 4 | 6 | 6 | 7 | 5 | 6 | 1 | 0 | 1 | 0 | 7 | 6 | 5 | 4 |
| 27 | 3 | 4 | 4 | 3 | 4 | 3 | 4 | 4 | 3 | 1 | 0 | 0 | 0 | 3 | 4 | 5 | 3 |
| 28 | 7 | 6 | 5 | 7 | 6 | 7 | 5 | 4 | 5 | 1 | 0 | 1 | 0 | 7 | 5 | 6 | 7 |
| 29 | 3 | 5 | 4 | 3 | 5 | 5 | 3 | 4 | 3 | 1 | 0 | 0 | 0 | 3 | 4 | 5 | 4 |
| 30 | 4 | 5 | 4 | 3 | 3 | 4 | 5 | 5 | 4 | 0 | 0 | 1 | 0 | 3 | 4 | 3 | 5 |
| 31 | 5 | 5 | 3 | 5 | 3 | 4 | 5 | 5 | 3 | 0 | 0 | 0 | 1 | 4 | 5 | 3 | 4 |
| 32 | 5 | 4 | 4 | 6 | 3 | 5 | 4 | 5 | 3 | 0 | 1 | 0 | 1 | 5 | 3 | 5 | 4 |
| 33 | 1 | 2 | 1 | 3 | 3 | 1 | 2 | 1 | 1 | 0 | 0 | 1 | 0 | 3 | 2 | 1 | 2 |
| 34 | 3 | 4 | 5 | 3 | 5 | 4 | 3 | 3 | 4 | 0 | 1 | 1 | 0 | 4 | 5 | 4 | 5 |
| 35 | 4 | 5 | 3 | 4 | 3 | 5 | 6 | 3 | 5 | 0 | 0 | 0 | 0 | 3 | 5 | 5 | 4 |
| 36 | 5 | 3 | 5 | 3 | 5 | 3 | 5 | 4 | 3 | 1 | 0 | 1 | 0 | 5 | 4 | 5 | 4 |
| 37 | 4 | 3 | 4 | 3 | 4 | 4 | 4 | 3 | 5 | 1 | 0 | 1 | 0 | 3 | 5 | 4 | 3 |
| 38 | 5 | 3 | 4 | 4 | 3 | 5 | 4 | 3 | 3 | 0 | 0 | 1 | 0 | 3 | 5 | 4 | 4 |
| 39 | 6 | 7 | 7 | 5 | 5 | 4 | 7 | 5 | 6 | 0 | 1 | 0 | 0 | 5 | 6 | 7 | 6 |
| 40 | 5 | 6 | 4 | 5 | 5 | 5 | 3 | 4 | 5 | 0 | 0 | 1 | 0 | 4 | 5 | 4 | 4 |
| 41 | 7 | 5 | 6 | 5 | 7 | 7 | 6 | 5 | 6 | 1 | 0 | 1 | 0 | 7 | 6 | 5 | 7 |
| 42 | 3 | 5 | 4 | 4 | 3 | 4 | 5 | 3 | 4 | 0 | 0 | 1 | 0 | 4 | 3 | 4 | 4 |
| 43 | 5 | 5 | 4 | 5 | 5 | 4 | 5 | 4 | 3 | 0 | 0 | 1 | 0 | 4 | 5 | 4 | 4 |
| 44 | 4 | 4 | 5 | 4 | 3 | 5 | 4 | 3 | 4 | 0 | 0 | 0 | 0 | 5 | 4 | 3 | 4 |
| 45 | 1 | 2 | 3 | 1 | 2 | 1 | 2 | 3 | 1 | 1 | 0 | 1 | 0 | 2 | 2 | 2 | 1 |
| 46 | 2 | 2 | 1 | 2 | 1 | 2 | 1 | 2 | 1 | 1 | 0 | 1 | 0 | 2 | 1 | 1 | 2 |
| 47 | 5 | 5 | 4 | 5 | 3 | 4 | 5 | 5 | 4 | 0 | 0 | 0 | 1 | 4 | 5 | 4 | 4 |
| 48 | 5 | 5 | 4 | 6 | 3 | 5 | 4 | 5 | 3 | 1 | 0 | 1 | 0 | 5 | 4 | 5 | 4 |
| 49 | 1 | 2 | 1 | 2 | 2 | 1 | 2 | 1 | 1 | 1 | 0 | 1 | 0 | 1 | 2 | 1 | 2 |
| 50 | 3 | 3 | 2 | 3 | 3 | 4 | 3 | 3 | 3 | 0 | 0 | 1 | 0 | 4 | 3 | 3 | 3 |
| 51 | 4 | 5 | 3 | 4 | 3 | 4 | 4 | 3 | 5 | 0 | 1 | 1 | 0 | 3 | 5 | 5 | 4 |
| 52 | 5 | 5 | 6 | 4 | 5 | 4 | 3 | 4 | 4 | 0 | 0 | 1 | 0 | 3 | 5 | 4 | 5 |
| 53 | 3 | 5 | 4 | 3 | 3 | 3 | 5 | 4 | 4 | 0 | 0 | 0 | 0 | 4 | 4 | 5 | 4 |
| 54 | 4 | 3 | 4 | 5 | 4 | 3 | 3 | 3 | 5 | 1 | 0 | 1 | 0 | 4 | 3 | 5 | 4 |
| 55 | 6 | 7 | 5 | 6 | 3 | 4 | 5 | 4 | 7 | 0 | 0 | 1 | 0 | 5 | 7 | 5 | 7 |
| 56 | 3 | 5 | 5 | 4 | 3 | 4 | 5 | 4 | 3 | 0 | 0 | 0 | 0 | 5 | 4 | 4 | 5 |
| 57 | 6 | 5 | 7 | 6 | 7 | 5 | 4 | 6 | 7 | 1 | 0 | 0 | 1 | 7 | 5 | 6 | 5 |
| 58 | 6 | 7 | 7 | 5 | 5 | 4 | 7 | 5 | 6 | 1 | 0 | 1 | 0 | 5 | 6 | 7 | 6 |
| 59 | 5 | 6 | 6 | 5 | 5 | 5 | 3 | 4 | 5 | 1 | 0 | 0 | 0 | 3 | 5 | 4 | 4 |
| 60 | 7 | 5 | 6 | 4 | 7 | 7 | 6 | 5 | 6 | 0 | 0 | 0 | 0 | 7 | 6 | 5 | 7 |
| 61 | 4 | 3 | 4 | 5 | 3 | 4 | 5 | 3 | 4 | 0 | 1 | 0 | 0 | 4 | 3 | 4 | 4 |
| 62 | 5 | 4 | 4 | 5 | 5 | 3 | 4 | 4 | 3 | 0 | 0 | 1 | 0 | 4 | 5 | 4 | 4 |
| 63 | 4 | 4 | 5 | 4 | 3 | 5 | 4 | 3 | 5 | 0 | 1 | 1 | 0 | 5 | 4 | 3 | 4 |
| 64 | 1 | 2 | 2 | 1 | 2 | 1 | 2 | 2 | 1 | 1 | 0 | 0 | 0 | 1 | 2 | 1 | 1 |
| 65 | 3 | 2 | 1 | 2 | 1 | 1 | 1 | 2 | 1 | 1 | 0 | 0 | 0 | 2 | 2 | 1 | 2 |
| 66 | 5 | 5 | 4 | 5 | 3 | 4 | 5 | 5 | 3 | 0 | 0 | 1 | 0 | 4 | 5 | 3 | 4 |
| 67 | 5 | 5 | 4 | 6 | 3 | 3 | 4 | 5 | 3 | 0 | 1 | 1 | 0 | 5 | 3 | 5 | 4 |
| 68 | 4 | 3 | 5 | 5 | 3 | 4 | 3 | 4 | 5 | 0 | 0 | 1 | 0 | 4 | 5 | 4 | 3 |
| 69 | 5 | 5 | 4 | 5 | 5 | 3 | 5 | 4 | 3 | 1 | 0 | 1 | 0 | 5 | 5 | 4 | 3 |
| 70 | 4 | 4 | 5 | 4 | 3 | 5 | 4 | 3 | 4 | 0 | 0 | 1 | 0 | 5 | 4 | 3 | 4 |
| 71 | 1 | 2 | 2 | 1 | 2 | 1 | 2 | 2 | 1 | 0 | 1 | 0 | 0 | 3 | 2 | 3 | 1 |
| 72 | 3 | 2 | 1 | 2 | 1 | 1 | 1 | 2 | 1 | 1 | 0 | 0 | 0 | 2 | 1 | 1 | 2 |
| 73 | 5 | 5 | 4 | 4 | 3 | 5 | 5 | 5 | 3 | 1 | 0 | 1 | 0 | 4 | 5 | 3 | 4 |
| 74 | 5 | 5 | 4 | 5 | 3 | 5 | 4 | 5 | 3 | 0 | 0 | 0 | 0 | 5 | 3 | 5 | 4 |
| 75 | 2 | 2 | 1 | 2 | 2 | 1 | 2 | 1 | 1 | 0 | 0 | 0 | 1 | 3 | 2 | 1 | 2 |
| 76 | 3 | 5 | 4 | 4 | 3 | 4 | 5 | 4 | 3 | 0 | 0 | 1 | 0 | 5 | 4 | 4 | 5 |
| 77 | 6 | 5 | 7 | 6 | 7 | 5 | 4 | 5 | 7 | 0 | 0 | 1 | 0 | 7 | 5 | 6 | 5 |
| 78 | 1 | 2 | 2 | 1 | 1 | 2 | 3 | 3 | 3 | 1 | 0 | 1 | 0 | 1 | 2 | 2 | 2 |
| 79 | 5 | 3 | 4 | 5 | 4 | 5 | 3 | 4 | 5 | 0 | 0 | 1 | 0 | 3 | 5 | 3 | 5 |
| 80 | 3 | 5 | 5 | 4 | 3 | 5 | 5 | 4 | 3 | 0 | 0 | 1 | 0 | 5 | 3 | 4 | 5 |
| 81 | 6 | 5 | 6 | 6 | 7 | 5 | 4 | 5 | 7 | 0 | 0 | 1 | 0 | 7 | 5 | 6 | 5 |
| 82 | 1 | 2 | 2 | 1 | 3 | 2 | 3 | 4 | 3 | 1 | 0 | 1 | 0 | 3 | 2 | 3 | 2 |
| 83 | 3 | 3 | 2 | 4 | 5 | 3 | 4 | 5 | 3 | 1 | 0 | 0 | 1 | 3 | 4 | 5 | 4 |
| 84 | 3 | 2 | 5 | 4 | 4 | 5 | 4 | 3 | 4 | 0 | 0 | 0 | 0 | 4 | 5 | 3 | 5 |
| 85 | 4 | 3 | 4 | 5 | 4 | 5 | 3 | 4 | 5 | 0 | 1 | 0 | 0 | 3 | 5 | 3 | 5 |
| 86 | 2 | 2 | 1 | 2 | 1 | 2 | 2 | 2 | 1 | 1 | 0 | 0 | 0 | 1 | 2 | 1 | 1 |
| 87 | 4 | 3 | 3 | 5 | 3 | 4 | 3 | 4 | 3 | 0 | 0 | 1 | 0 | 4 | 3 | 3 | 3 |
| 88 | 3 | 5 | 4 | 3 | 6 | 3 | 4 | 3 | 5 | 0 | 0 | 0 | 0 | 3 | 4 | 5 | 4 |
| 89 | 4 | 4 | 3 | 5 | 3 | 4 | 3 | 4 | 4 | 1 | 0 | 0 | 1 | 4 | 5 | 4 | 3 |
| 90 | 3 | 2 | 3 | 4 | 2 | 3 | 4 | 3 | 5 | 0 | 0 | 1 | 0 | 4 | 4 | 3 | 4 |
| 91 | 1 | 1 | 2 | 3 | 2 | 1 | 2 | 1 | 1 | 0 | 1 | 0 | 0 | 2 | 1 | 1 | 2 |
| 92 | 4 | 3 | 5 | 5 | 3 | 4 | 5 | 3 | 5 | 0 | 0 | 1 | 0 | 3 | 4 | 5 | 3 |
| 93 | 5 | 5 | 4 | 5 | 5 | 4 | 3 | 4 | 5 | 1 | 0 | 1 | 0 | 3 | 4 | 5 | 3 |
| 94 | 4 | 3 | 5 | 4 | 4 | 5 | 4 | 4 | 3 | 1 | 0 | 0 | 0 | 5 | 3 | 3 | 4 |
| 95 | 6 | 5 | 6 | 7 | 3 | 7 | 5 | 6 | 5 | 1 | 0 | 1 | 0 | 7 | 5 | 7 | 6 |
| 96 | 3 | 2 | 3 | 4 | 3 | 3 | 3 | 2 | 3 | 0 | 0 | 1 | 0 | 4 | 3 | 4 | 5 |
| 97 | 3 | 5 | 3 | 4 | 3 | 5 | 3 | 4 | 3 | 0 | 1 | 1 | 0 | 5 | 3 | 4 | 3 |
| 98 | 3 | 3 | 4 | 3 | 4 | 3 | 4 | 4 | 4 | 0 | 0 | 0 | 0 | 3 | 4 | 3 | 4 |
| 99 | 2 | 2 | 2 | 3 | 2 | 3 | 3 | 3 | 2 | 0 | 0 | 1 | 0 | 2 | 3 | 2 | 2 |
| 100 | 3 | 2 | 2 | 3 | 4 | 5 | 3 | 4 | 5 | 1 | 0 | 1 | 0 | 3 | 4 | 3 | 4 |
| 101 | 2 | 2 | 1 | 2 | 1 | 1 | 1 | 2 | 1 | 1 | 0 | 1 | 0 | 2 | 1 | 1 | 2 |
| 102 | 5 | 5 | 4 | 5 | 5 | 4 | 5 | 5 | 3 | 0 | 0 | 0 | 1 | 4 | 5 | 3 | 4 |
| 103 | 4 | 3 | 4 | 5 | 4 | 3 | 3 | 3 | 5 | 1 | 0 | 1 | 0 | 4 | 3 | 5 | 4 |
| 104 | 6 | 7 | 5 | 6 | 3 | 4 | 5 | 4 | 7 | 0 | 0 | 0 | 0 | 5 | 7 | 5 | 7 |
| 105 | 4 | 5 | 5 | 4 | 3 | 4 | 5 | 4 | 3 | 1 | 0 | 1 | 0 | 5 | 3 | 4 | 5 |
| 106 | 6 | 5 | 7 | 6 | 7 | 5 | 4 | 5 | 5 | 0 | 0 | 1 | 0 | 7 | 5 | 6 | 5 |
| 107 | 6 | 7 | 7 | 5 | 6 | 4 | 5 | 5 | 6 | 0 | 1 | 1 | 0 | 5 | 6 | 7 | 6 |
| 108 | 5 | 5 | 4 | 5 | 5 | 5 | 3 | 4 | 5 | 0 | 0 | 1 | 0 | 3 | 5 | 4 | 4 |
| 109 | 6 | 5 | 6 | 4 | 7 | 7 | 6 | 5 | 7 | 1 | 0 | 1 | 0 | 7 | 6 | 5 | 7 |
| 110 | 3 | 5 | 4 | 5 | 3 | 4 | 5 | 3 | 4 | 0 | 0 | 0 | 0 | 4 | 3 | 4 | 4 |
| 111 | 5 | 5 | 4 | 5 | 4 | 3 | 5 | 4 | 3 | 0 | 0 | 0 | 1 | 4 | 5 | 4 | 3 |
| 112 | 4 | 4 | 5 | 3 | 3 | 5 | 4 | 3 | 4 | 1 | 0 | 1 | 0 | 5 | 4 | 3 | 4 |
| 113 | 6 | 7 | 5 | 5 | 6 | 4 | 7 | 5 | 6 | 0 | 0 | 1 | 0 | 5 | 6 | 7 | 6 |
| 114 | 5 | 5 | 4 | 5 | 5 | 5 | 3 | 4 | 5 | 0 | 0 | 1 | 0 | 3 | 5 | 4 | 4 |
| 115 | 6 | 5 | 6 | 4 | 7 | 7 | 6 | 5 | 7 | 1 | 0 | 1 | 0 | 7 | 6 | 5 | 7 |
| 116 | 4 | 5 | 4 | 4 | 3 | 4 | 4 | 3 | 4 | 0 | 1 | 0 | 0 | 4 | 3 | 4 | 4 |
| 117 | 5 | 4 | 4 | 5 | 5 | 3 | 5 | 4 | 3 | 1 | 0 | 1 | 0 | 4 | 5 | 4 | 3 |
| 118 | 3 | 4 | 5 | 4 | 3 | 5 | 4 | 3 | 4 | 1 | 0 | 1 | 0 | 5 | 4 | 3 | 4 |
| 119 | 1 | 2 | 2 | 1 | 2 | 1 | 2 | 2 | 1 | 1 | 0 | 1 | 0 | 3 | 2 | 3 | 1 |
| 120 | 2 | 2 | 1 | 2 | 1 | 1 | 1 | 2 | 1 | 0 | 0 | 1 | 0 | 2 | 1 | 1 | 2 |
| 121 | 5 | 5 | 4 | 5 | 3 | 3 | 5 | 5 | 3 | 0 | 1 | 1 | 0 | 4 | 5 | 3 | 4 |
| 122 | 5 | 5 | 4 | 6 | 3 | 3 | 4 | 5 | 3 | 0 | 0 | 0 | 0 | 5 | 3 | 5 | 4 |
| 123 | 4 | 5 | 5 | 4 | 3 | 4 | 5 | 4 | 3 | 1 | 0 | 1 | 0 | 5 | 3 | 4 | 5 |
| 124 | 5 | 5 | 7 | 6 | 7 | 5 | 4 | 5 | 7 | 1 | 0 | 1 | 0 | 7 | 5 | 6 | 5 |
| 125 | 1 | 2 | 2 | 1 | 2 | 2 | 3 | 2 | 3 | 1 | 0 | 1 | 0 | 3 | 2 | 3 | 2 |
| 126 | 4 | 2 | 2 | 4 | 5 | 3 | 4 | 5 | 3 | 1 | 0 | 1 | 0 | 3 | 4 | 5 | 4 |
| 127 | 4 | 2 | 5 | 4 | 3 | 5 | 4 | 3 | 4 | 0 | 0 | 1 | 0 | 4 | 5 | 3 | 5 |
| 128 | 5 | 4 | 4 | 5 | 4 | 5 | 3 | 4 | 5 | 0 | 0 | 0 | 1 | 4 | 5 | 3 | 5 |
| 129 | 1 | 2 | 1 | 2 | 1 | 2 | 3 | 2 | 1 | 0 | 0 | 0 | 0 | 1 | 2 | 2 | 2 |
| 130 | 4 | 4 | 3 | 5 | 3 | 4 | 3 | 4 | 3 | 0 | 1 | 1 | 0 | 4 | 3 | 3 | 3 |
| 131 | 5 | 3 | 4 | 3 | 6 | 3 | 4 | 3 | 5 | 1 | 0 | 0 | 0 | 3 | 4 | 5 | 4 |
| 132 | 3 | 4 | 3 | 5 | 3 | 4 | 3 | 4 | 5 | 1 | 0 | 1 | 0 | 4 | 5 | 4 | 3 |
| 133 | 3 | 2 | 3 | 3 | 2 | 3 | 4 | 3 | 5 | 1 | 0 | 1 | 0 | 4 | 5 | 3 | 4 |
| 134 | 1 | 2 | 2 | 3 | 2 | 1 | 2 | 1 | 1 | 0 | 0 | 1 | 0 | 2 | 1 | 1 | 2 |
| 135 | 3 | 3 | 4 | 5 | 3 | 4 | 5 | 3 | 5 | 0 | 0 | 1 | 0 | 3 | 4 | 5 | 3 |
| 136 | 5 | 4 | 5 | 3 | 4 | 5 | 3 | 4 | 5 | 1 | 0 | 0 | 0 | 5 | 5 | 4 | 3 |
| 137 | 5 | 5 | 6 | 5 | 3 | 3 | 4 | 5 | 4 | 1 | 0 | 1 | 0 | 3 | 5 | 4 | 4 |
| 138 | 6 | 5 | 4 | 5 | 5 | 4 | 3 | 5 | 4 | 1 | 0 | 1 | 0 | 5 | 6 | 5 | 5 |
| 139 | 3 | 4 | 3 | 4 | 3 | 4 | 5 | 4 | 3 | 0 | 0 | 0 | 1 | 3 | 4 | 4 | 3 |
| 140 | 3 | 3 | 2 | 4 | 2 | 3 | 3 | 3 | 2 | 0 | 1 | 0 | 0 | 2 | 3 | 2 | 2 |
| 141 | 3 | 2 | 3 | 3 | 4 | 5 | 3 | 4 | 5 | 0 | 0 | 1 | 0 | 4 | 4 | 3 | 4 |
| 142 | 5 | 4 | 5 | 5 | 4 | 5 | 4 | 3 | 4 | 0 | 0 | 1 | 0 | 4 | 5 | 3 | 5 |
| 143 | 4 | 3 | 4 | 3 | 5 | 4 | 3 | 4 | 4 | 0 | 0 | 0 | 1 | 5 | 4 | 5 | 4 |
| 144 | 4 | 4 | 5 | 3 | 4 | 3 | 5 | 4 | 3 | 0 | 0 | 1 | 0 | 3 | 5 | 4 | 4 |
| 145 | 4 | 4 | 5 | 3 | 4 | 3 | 5 | 4 | 3 | 1 | 0 | 1 | 0 | 5 | 4 | 4 | 5 |
| 146 | 5 | 5 | 4 | 3 | 4 | 5 | 3 | 4 | 5 | 0 | 0 | 1 | 0 | 3 | 4 | 3 | 4 |
| 147 | 3 | 2 | 3 | 4 | 2 | 3 | 4 | 3 | 4 | 0 | 0 | 1 | 0 | 4 | 5 | 3 | 4 |
| 148 | 1 | 1 | 2 | 1 | 2 | 2 | 2 | 1 | 1 | 0 | 1 | 0 | 0 | 2 | 1 | 1 | 2 |
| 149 | 4 | 3 | 4 | 5 | 3 | 3 | 5 | 3 | 5 | 0 | 0 | 1 | 0 | 3 | 4 | 5 | 3 |
| 150 | 5 | 6 | 4 | 5 | 4 | 4 | 3 | 4 | 5 | 1 | 0 | 1 | 0 | 3 | 4 | 5 | 3 |
| 151 | 4 | 4 | 5 | 4 | 3 | 5 | 3 | 4 | 3 | 0 | 0 | 0 | 0 | 5 | 3 | 3 | 4 |
| 152 | 5 | 5 | 7 | 7 | 3 | 7 | 5 | 6 | 5 | 1 | 0 | 1 | 0 | 7 | 5 | 7 | 6 |
| 153 | 5 | 6 | 5 | 4 | 4 | 5 | 4 | 4 | 3 | 0 | 0 | 1 | 0 | 4 | 3 | 4 | 5 |
| 154 | 6 | 5 | 5 | 4 | 7 | 6 | 7 | 5 | 6 | 1 | 0 | 0 | 0 | 7 | 6 | 5 | 4 |
| 155 | 3 | 4 | 5 | 3 | 4 | 3 | 4 | 4 | 3 | 1 | 0 | 1 | 0 | 3 | 4 | 5 | 3 |
| 156 | 5 | 6 | 5 | 7 | 6 | 7 | 5 | 4 | 5 | 0 | 0 | 1 | 0 | 7 | 5 | 6 | 7 |
| 157 | 3 | 5 | 3 | 3 | 5 | 5 | 3 | 4 | 3 | 0 | 0 | 1 | 0 | 3 | 4 | 5 | 4 |
| 158 | 5 | 5 | 4 | 5 | 3 | 4 | 5 | 5 | 4 | 0 | 1 | 1 | 0 | 3 | 4 | 3 | 5 |
| 159 | 5 | 5 | 4 | 5 | 3 | 4 | 5 | 5 | 4 | 0 | 0 | 0 | 0 | 4 | 5 | 3 | 4 |
| 160 | 5 | 5 | 4 | 5 | 3 | 5 | 4 | 3 | 3 | 1 | 0 | 1 | 0 | 5 | 3 | 5 | 4 |
| 161 | 7 | 5 | 6 | 4 | 5 | 7 | 6 | 5 | 7 | 1 | 0 | 1 | 0 | 7 | 6 | 5 | 7 |
| 162 | 3 | 5 | 4 | 5 | 3 | 4 | 5 | 3 | 4 | 0 | 0 | 1 | 0 | 4 | 3 | 4 | 4 |
| 163 | 5 | 4 | 4 | 5 | 5 | 3 | 5 | 4 | 3 | 0 | 0 | 1 | 0 | 4 | 5 | 4 | 3 |
| 164 | 5 | 4 | 5 | 4 | 3 | 5 | 4 | 3 | 4 | 0 | 1 | 0 | 0 | 5 | 4 | 3 | 4 |
| 165 | 1 | 2 | 2 | 1 | 2 | 1 | 2 | 2 | 1 | 0 | 0 | 1 | 0 | 3 | 2 | 3 | 1 |
| 166 | 3 | 1 | 1 | 2 | 1 | 2 | 1 | 2 | 1 | 0 | 0 | 0 | 1 | 2 | 1 | 1 | 2 |
| 167 | 4 | 5 | 5 | 4 | 5 | 3 | 4 | 5 | 4 | 1 | 0 | 1 | 0 | 3 | 5 | 4 | 4 |
| 168 | 3 | 4 | 5 | 3 | 4 | 5 | 3 | 4 | 5 | 0 | 0 | 1 | 0 | 5 | 5 | 3 | 3 |
| 169 | 5 | 5 | 6 | 5 | 4 | 3 | 4 | 5 | 3 | 0 | 0 | 1 | 0 | 3 | 5 | 4 | 4 |
| 170 | 1 | 2 | 3 | 2 | 1 | 3 | 3 | 1 | 1 | 0 | 1 | 0 | 0 | 1 | 2 | 1 | 2 |
| 171 | 6 | 7 | 5 | 7 | 7 | 5 | 6 | 6 | 7 | 0 | 0 | 1 | 0 | 7 | 5 | 6 | 7 |
| 172 | 4 | 6 | 5 | 5 | 5 | 4 | 6 | 5 | 5 | 0 | 0 | 1 | 0 | 6 | 5 | 6 | 4 |
| 173 | 3 | 4 | 4 | 6 | 5 | 6 | 5 | 5 | 6 | 0 | 0 | 1 | 0 | 6 | 4 | 4 | 6 |
| 174 | 3 | 3 | 4 | 4 | 6 | 5 | 6 | 3 | 4 | 1 | 0 | 1 | 0 | 5 | 5 | 3 | 6 |
| 175 | 4 | 5 | 4 | 7 | 5 | 7 | 6 | 5 | 3 | 1 | 0 | 0 | 0 | 7 | 7 | 5 | 6 |
| 176 | 5 | 5 | 4 | 3 | 5 | 7 | 6 | 5 | 6 | 1 | 0 | 1 | 0 | 4 | 5 | 6 | 6 |
| 177 | 3 | 2 | 3 | 2 | 3 | 2 | 1 | 3 | 3 | 0 | 0 | 1 | 0 | 2 | 3 | 3 | 2 |
| 178 | 5 | 6 | 3 | 5 | 4 | 6 | 3 | 5 | 6 | 0 | 0 | 1 | 0 | 3 | 5 | 5 | 4 |
| 179 | 3 | 2 | 5 | 6 | 4 | 2 | 3 | 4 | 5 | 0 | 1 | 1 | 0 | 5 | 2 | 4 | 5 |
| 180 | 2 | 1 | 1 | 2 | 1 | 1 | 2 | 3 | 1 | 1 | 0 | 0 | 1 | 3 | 3 | 3 | 3 |
| 181 | 3 | 4 | 5 | 5 | 4 | 4 | 5 | 4 | 4 | 1 | 0 | 0 | 1 | 5 | 3 | 5 | 4 |
| 182 | 4 | 5 | 3 | 5 | 4 | 5 | 3 | 5 | 5 | 0 | 0 | 0 | 1 | 5 | 3 | 5 | 4 |
| 183 | 3 | 3 | 3 | 4 | 3 | 4 | 3 | 2 | 3 | 0 | 0 | 1 | 0 | 4 | 3 | 4 | 5 |
| 184 | 3 | 5 | 3 | 4 | 3 | 5 | 3 | 4 | 3 | 0 | 1 | 1 | 0 | 5 | 3 | 4 | 3 |
| 185 | 3 | 5 | 4 | 3 | 4 | 3 | 4 | 5 | 4 | 0 | 0 | 0 | 0 | 3 | 4 | 3 | 4 |
| 186 | 2 | 3 | 2 | 4 | 3 | 3 | 3 | 3 | 2 | 0 | 0 | 1 | 0 | 2 | 3 | 2 | 2 |
| 187 | 3 | 4 | 2 | 3 | 4 | 5 | 3 | 4 | 5 | 1 | 0 | 1 | 0 | 4 | 4 | 3 | 4 |
| 188 | 4 | 5 | 4 | 5 | 3 | 5 | 4 | 3 | 4 | 1 | 0 | 1 | 0 | 4 | 5 | 3 | 5 |
| 189 | 4 | 3 | 4 | 3 | 5 | 4 | 3 | 4 | 5 | 0 | 0 | 0 | 1 | 5 | 4 | 5 | 4 |
| 190 | 3 | 4 | 5 | 3 | 4 | 3 | 4 | 4 | 3 | 0 | 1 | 1 | 0 | 5 | 5 | 4 | 4 |
| 191 | 4 | 5 | 5 | 3 | 4 | 3 | 5 | 4 | 3 | 0 | 0 | 1 | 0 | 5 | 4 | 3 | 5 |
| 192 | 4 | 5 | 4 | 3 | 4 | 5 | 3 | 4 | 5 | 0 | 0 | 1 | 0 | 5 | 4 | 3 | 4 |
| 193 | 5 | 4 | 5 | 3 | 5 | 5 | 4 | 5 | 3 | 0 | 0 | 1 | 0 | 4 | 5 | 5 | 4 |
| 194 | 4 | 5 | 3 | 4 | 4 | 5 | 3 | 4 | 3 | 0 | 0 | 0 | 0 | 3 | 3 | 4 | 3 |
| 195 | 3 | 5 | 4 | 4 | 5 | 3 | 4 | 5 | 4 | 1 | 0 | 0 | 0 | 3 | 4 | 3 | 4 |
| 196 | 2 | 3 | 2 | 4 | 2 | 3 | 3 | 3 | 3 | 0 | 0 | 1 | 0 | 2 | 3 | 2 | 2 |
| 197 | 5 | 7 | 5 | 6 | 3 | 4 | 5 | 4 | 7 | 0 | 0 | 1 | 0 | 5 | 7 | 5 | 7 |
| 198 | 3 | 5 | 5 | 4 | 3 | 4 | 5 | 4 | 3 | 0 | 1 | 1 | 0 | 5 | 3 | 4 | 5 |
| 199 | 6 | 5 | 7 | 6 | 7 | 5 | 4 | 5 | 7 | 0 | 0 | 0 | 1 | 7 | 5 | 6 | 5 |
| 200 | 1 | 2 | 2 | 1 | 3 | 2 | 2 | 4 | 3 | 0 | 0 | 1 | 0 | 3 | 2 | 3 | 2 |
| 201 | 4 | 3 | 2 | 4 | 3 | 3 | 4 | 5 | 3 | 0 | 1 | 1 | 0 | 3 | 4 | 5 | 4 |
| 202 | 3 | 2 | 5 | 4 | 3 | 5 | 4 | 3 | 4 | 0 | 0 | 1 | 0 | 4 | 5 | 3 | 5 |
| 203 | 5 | 3 | 4 | 5 | 4 | 5 | 3 | 4 | 4 | 0 | 0 | 0 | 0 | 3 | 5 | 3 | 5 |
| 204 | 2 | 2 | 1 | 2 | 1 | 2 | 3 | 2 | 1 | 1 | 0 | 1 | 0 | 1 | 2 | 3 | 3 |
| 205 | 3 | 4 | 3 | 4 | 3 | 4 | 3 | 4 | 3 | 0 | 0 | 1 | 0 | 4 | 3 | 3 | 3 |
| 206 | 5 | 5 | 4 | 3 | 6 | 5 | 4 | 3 | 5 | 1 | 0 | 1 | 0 | 3 | 4 | 5 | 4 |
| 207 | 4 | 4 | 3 | 5 | 3 | 4 | 3 | 4 | 5 | 0 | 0 | 1 | 0 | 4 | 5 | 4 | 3 |
| 208 | 3 | 2 | 3 | 4 | 2 | 3 | 4 | 3 | 4 | 1 | 0 | 1 | 0 | 4 | 4 | 3 | 4 |
| 209 | 1 | 1 | 2 | 3 | 2 | 1 | 2 | 2 | 1 | 0 | 0 | 1 | 0 | 2 | 1 | 1 | 2 |
| 210 | 4 | 3 | 4 | 5 | 3 | 4 | 5 | 3 | 5 | 0 | 1 | 1 | 0 | 3 | 4 | 5 | 3 |
| 211 | 4 | 5 | 4 | 5 | 4 | 3 | 3 | 3 | 5 | 0 | 0 | 1 | 0 | 4 | 3 | 5 | 4 |
| 212 | 6 | 5 | 6 | 5 | 7 | 5 | 4 | 5 | 7 | 1 | 0 | 0 | 1 | 7 | 5 | 6 | 5 |
| 213 | 1 | 2 | 2 | 1 | 3 | 2 | 3 | 4 | 3 | 1 | 0 | 1 | 0 | 1 | 2 | 1 | 2 |
| 214 | 4 | 4 | 3 | 4 | 5 | 3 | 4 | 5 | 3 | 0 | 0 | 1 | 0 | 3 | 4 | 5 | 4 |
| 215 | 3 | 4 | 5 | 4 | 3 | 5 | 4 | 3 | 4 | 0 | 0 | 1 | 0 | 4 | 5 | 3 | 5 |
| 216 | 5 | 3 | 4 | 5 | 4 | 5 | 3 | 4 | 5 | 0 | 0 | 1 | 0 | 3 | 5 | 3 | 5 |
| 217 | 2 | 2 | 1 | 2 | 1 | 2 | 3 | 2 | 1 | 0 | 0 | 1 | 0 | 1 | 2 | 3 | 3 |
| 218 | 4 | 4 | 3 | 5 | 3 | 4 | 3 | 4 | 3 | 1 | 0 | 0 | 0 | 4 | 3 | 3 | 3 |
| 219 | 5 | 5 | 4 | 3 | 6 | 3 | 4 | 3 | 5 | 1 | 0 | 1 | 0 | 3 | 4 | 5 | 4 |
| 220 | 4 | 5 | 3 | 5 | 3 | 4 | 3 | 4 | 5 | 0 | 0 | 1 | 0 | 4 | 5 | 4 | 3 |
| 221 | 3 | 2 | 3 | 4 | 2 | 3 | 4 | 3 | 5 | 1 | 0 | 0 | 0 | 4 | 5 | 3 | 4 |
| 222 | 1 | 1 | 2 | 3 | 2 | 1 | 2 | 1 | 1 | 0 | 0 | 1 | 0 | 2 | 1 | 1 | 2 |
| 223 | 4 | 3 | 4 | 5 | 3 | 4 | 5 | 3 | 5 | 0 | 0 | 1 | 0 | 3 | 4 | 5 | 3 |
| 224 | 5 | 4 | 6 | 4 | 5 | 4 | 3 | 5 | 3 | 0 | 0 | 1 | 0 | 3 | 5 | 4 | 5 |
| 225 | 3 | 5 | 4 | 3 | 3 | 2 | 5 | 1 | 2 | 0 | 0 | 1 | 0 | 3 | 2 | 2 | 3 |
| 226 | 4 | 5 | 4 | 5 | 4 | 3 | 3 | 3 | 5 | 1 | 0 | 1 | 0 | 4 | 3 | 5 | 4 |
| 227 | 6 | 5 | 5 | 6 | 7 | 5 | 4 | 5 | 7 | 0 | 0 | 0 | 0 | 7 | 5 | 6 | 5 |
| 228 | 1 | 2 | 3 | 1 | 3 | 2 | 3 | 4 | 3 | 0 | 1 | 0 | 1 | 3 | 2 | 3 | 2 |
| 229 | 3 | 2 | 1 | 2 | 1 | 1 | 1 | 2 | 1 | 0 | 0 | 0 | 0 | 2 | 1 | 1 | 2 |
| 230 | 5 | 6 | 4 | 5 | 3 | 4 | 5 | 5 | 3 | 0 | 0 | 1 | 0 | 4 | 5 | 3 | 4 |
| 231 | 5 | 5 | 4 | 6 | 3 | 5 | 4 | 5 | 3 | 1 | 0 | 1 | 0 | 5 | 3 | 5 | 4 |
| 232 | 1 | 2 | 1 | 3 | 2 | 1 | 2 | 1 | 1 | 1 | 0 | 1 | 0 | 3 | 2 | 1 | 2 |
| 233 | 3 | 4 | 2 | 3 | 5 | 4 | 3 | 3 | 3 | 0 | 0 | 0 | 0 | 4 | 3 | 4 | 5 |
| 234 | 4 | 5 | 3 | 4 | 3 | 5 | 4 | 3 | 5 | 1 | 0 | 1 | 0 | 3 | 5 | 5 | 4 |
| 235 | 3 | 2 | 3 | 4 | 3 | 2 | 4 | 3 | 5 | 0 | 0 | 1 | 0 | 3 | 4 | 3 | 3 |
| 236 | 7 | 6 | 7 | 7 | 5 | 7 | 6 | 7 | 4 | 0 | 1 | 1 | 0 | 4 | 7 | 6 | 5 |
| 237 | 5 | 5 | 4 | 5 | 6 | 4 | 3 | 5 | 4 | 0 | 0 | 0 | 0 | 4 | 3 | 5 | 4 |
| 238 | 4 | 4 | 5 | 3 | 3 | 3 | 3 | 5 | 5 | 0 | 1 | 1 | 0 | 5 | 4 | 4 | 5 |
| 239 | 4 | 3 | 5 | 4 | 5 | 3 | 5 | 4 | 3 | 0 | 0 | 0 | 1 | 5 | 4 | 5 | 4 |
| 240 | 3 | 2 | 3 | 4 | 2 | 3 | 4 | 4 | 5 | 1 | 0 | 0 | 0 | 4 | 5 | 3 | 4 |
| 241 | 1 | 1 | 2 | 3 | 2 | 1 | 2 | 1 | 1 | 0 | 0 | 1 | 0 | 2 | 1 | 1 | 2 |
| 242 | 4 | 4 | 4 | 5 | 3 | 4 | 5 | 3 | 5 | 0 | 0 | 1 | 0 | 3 | 4 | 5 | 3 |
| 243 | 5 | 6 | 4 | 5 | 5 | 4 | 3 | 4 | 5 | 0 | 0 | 0 | 0 | 3 | 4 | 5 | 3 |
| 244 | 4 | 3 | 5 | 4 | 3 | 5 | 3 | 5 | 3 | 0 | 0 | 1 | 0 | 5 | 3 | 3 | 4 |
| 245 | 7 | 5 | 7 | 7 | 6 | 5 | 5 | 6 | 5 | 0 | 0 | 1 | 0 | 7 | 5 | 7 | 6 |
| 246 | 3 | 2 | 3 | 4 | 3 | 4 | 3 | 2 | 3 | 0 | 0 | 1 | 0 | 4 | 3 | 4 | 5 |
| 247 | 4 | 5 | 3 | 4 | 3 | 5 | 3 | 4 | 3 | 0 | 0 | 1 | 0 | 5 | 3 | 4 | 3 |
| 248 | 3 | 5 | 4 | 3 | 5 | 3 | 5 | 5 | 4 | 0 | 0 | 1 | 0 | 3 | 4 | 3 | 4 |
| 249 | 2 | 3 | 2 | 4 | 2 | 3 | 3 | 3 | 4 | 0 | 0 | 0 | 0 | 2 | 3 | 2 | 2 |
| 250 | 3 | 2 | 2 | 3 | 4 | 5 | 3 | 4 | 5 | 0 | 1 | 1 | 0 | 4 | 4 | 3 | 4 |
| 251 | 5 | 5 | 4 | 5 | 3 | 5 | 4 | 3 | 4 | 0 | 0 | 1 | 0 | 4 | 5 | 3 | 5 |
| 252 | 4 | 3 | 4 | 3 | 5 | 4 | 3 | 4 | 5 | 0 | 0 | 0 | 0 | 5 | 4 | 5 | 4 |
| 253 | 3 | 4 | 5 | 3 | 4 | 3 | 5 | 4 | 3 | 0 | 0 | 1 | 0 | 5 | 5 | 4 | 4 |
| 254 | 4 | 4 | 5 | 3 | 4 | 3 | 4 | 4 | 3 | 0 | 0 | 1 | 0 | 5 | 4 | 3 | 5 |
| 255 | 5 | 5 | 4 | 3 | 4 | 5 | 3 | 4 | 5 | 0 | 0 | 1 | 0 | 5 | 4 | 3 | 4 |
| 256 | 5 | 4 | 5 | 3 | 4 | 5 | 4 | 5 | 3 | 0 | 0 | 1 | 0 | 4 | 5 | 5 | 4 |
| 257 | 5 | 3 | 4 | 5 | 3 | 4 | 5 | 6 | 6 | 1 | 0 | 0 | 1 | 5 | 4 | 6 | 6 |
| 258 | 4 | 5 | 4 | 5 | 4 | 5 | 5 | 4 | 3 | 0 | 0 | 0 | 0 | 4 | 5 | 6 | 4 |
| 259 | 5 | 5 | 4 | 5 | 4 | 5 | 3 | 4 | 5 | 0 | 1 | 1 | 0 | 4 | 5 | 3 | 5 |
| 260 | 1 | 1 | 2 | 1 | 1 | 3 | 1 | 3 | 1 | 0 | 0 | 0 | 0 | 1 | 1 | 2 | 2 |
| 261 | 3 | 4 | 5 | 3 | 3 | 4 | 3 | 4 | 5 | 1 | 0 | 1 | 0 | 5 | 3 | 4 | 5 |
| 262 | 2 | 3 | 4 | 3 | 2 | 3 | 2 | 3 | 4 | 0 | 0 | 1 | 0 | 3 | 4 | 2 | 3 |
| 263 | 5 | 6 | 5 | 4 | 3 | 5 | 4 | 5 | 3 | 0 | 1 | 1 | 0 | 4 | 3 | 4 | 5 |
| 264 | 4 | 3 | 5 | 4 | 5 | 5 | 4 | 3 | 5 | 0 | 0 | 1 | 0 | 5 | 4 | 5 | 3 |
| 265 | 6 | 7 | 5 | 6 | 7 | 6 | 7 | 7 | 6 | 0 | 0 | 0 | 0 | 5 | 7 | 6 | 6 |
| 266 | 4 | 3 | 5 | 4 | 4 | 3 | 5 | 3 | 5 | 1 | 0 | 1 | 0 | 5 | 3 | 4 | 5 |
| 267 | 3 | 5 | 5 | 4 | 5 | 3 | 4 | 5 | 4 | 0 | 0 | 1 | 0 | 4 | 4 | 5 | 4 |
| 268 | 3 | 1 | 3 | 4 | 2 | 1 | 2 | 1 | 2 | 0 | 0 | 1 | 0 | 1 | 2 | 3 | 2 |
| 269 | 4 | 2 | 5 | 4 | 5 | 3 | 3 | 5 | 4 | 0 | 1 | 1 | 0 | 5 | 3 | 5 | 4 |
| 270 | 3 | 2 | 3 | 4 | 3 | 2 | 4 | 3 | 3 | 0 | 0 | 0 | 0 | 3 | 4 | 3 | 3 |
| 271 | 7 | 6 | 7 | 6 | 5 | 7 | 6 | 7 | 7 | 1 | 0 | 1 | 0 | 6 | 7 | 6 | 5 |
| 272 | 5 | 5 | 4 | 5 | 6 | 4 | 3 | 5 | 4 | 0 | 0 | 1 | 0 | 4 | 3 | 5 | 4 |
| 273 | 4 | 4 | 5 | 3 | 3 | 3 | 3 | 5 | 5 | 1 | 0 | 0 | 0 | 5 | 4 | 4 | 5 |
| 274 | 4 | 3 | 5 | 4 | 5 | 3 | 5 | 4 | 3 | 0 | 0 | 0 | 1 | 5 | 4 | 5 | 4 |
| 275 | 4 | 5 | 4 | 3 | 4 | 4 | 4 | 3 | 5 | 0 | 0 | 1 | 0 | 3 | 5 | 4 | 3 |
| 276 | 3 | 5 | 4 | 4 | 3 | 5 | 4 | 3 | 3 | 0 | 1 | 1 | 0 | 3 | 5 | 4 | 4 |
| 277 | 6 | 7 | 6 | 5 | 6 | 4 | 7 | 5 | 6 | 0 | 0 | 0 | 0 | 5 | 6 | 7 | 6 |
| 278 | 5 | 5 | 4 | 5 | 5 | 5 | 3 | 4 | 5 | 0 | 0 | 1 | 0 | 3 | 5 | 4 | 4 |
| 279 | 7 | 5 | 6 | 4 | 7 | 7 | 6 | 5 | 7 | 1 | 0 | 1 | 0 | 7 | 6 | 5 | 7 |
| 280 | 4 | 5 | 4 | 5 | 3 | 4 | 5 | 4 | 4 | 0 | 0 | 1 | 0 | 4 | 3 | 4 | 4 |
| 281 | 4 | 5 | 4 | 5 | 4 | 3 | 5 | 4 | 3 | 0 | 1 | 0 | 0 | 4 | 5 | 4 | 3 |
| 282 | 4 | 3 | 5 | 4 | 3 | 5 | 4 | 3 | 4 | 1 | 0 | 1 | 0 | 5 | 4 | 3 | 4 |
| 283 | 1 | 2 | 2 | 1 | 2 | 1 | 2 | 2 | 1 | 1 | 0 | 0 | 1 | 1 | 2 | 1 | 1 |
| 284 | 3 | 2 | 1 | 2 | 1 | 1 | 1 | 2 | 1 | 0 | 0 | 0 | 1 | 2 | 1 | 1 | 2 |
| 285 | 4 | 5 | 5 | 4 | 5 | 3 | 4 | 5 | 4 | 0 | 0 | 0 | 1 | 3 | 5 | 4 | 3 |
| 286 | 3 | 4 | 4 | 3 | 4 | 5 | 3 | 4 | 5 | 0 | 0 | 1 | 0 | 5 | 5 | 4 | 3 |
| 287 | 5 | 5 | 6 | 5 | 4 | 3 | 4 | 5 | 4 | 0 | 1 | 1 | 0 | 3 | 5 | 4 | 4 |
| 288 | 6 | 5 | 6 | 5 | 5 | 4 | 3 | 4 | 4 | 0 | 0 | 1 | 0 | 5 | 6 | 5 | 5 |
| 289 | 3 | 1 | 2 | 2 | 2 | 3 | 1 | 3 | 2 | 0 | 1 | 1 | 0 | 2 | 1 | 1 | 1 |
| 290 | 6 | 5 | 5 | 7 | 4 | 7 | 5 | 6 | 5 | 0 | 0 | 1 | 0 | 7 | 5 | 7 | 6 |
| 291 | 3 | 2 | 3 | 4 | 3 | 4 | 3 | 2 | 3 | 1 | 0 | 0 | 0 | 4 | 3 | 4 | 5 |
| 292 | 4 | 5 | 3 | 4 | 3 | 5 | 3 | 4 | 3 | 1 | 0 | 1 | 0 | 5 | 3 | 4 | 3 |
| 293 | 3 | 5 | 4 | 3 | 5 | 3 | 4 | 5 | 4 | 0 | 0 | 1 | 0 | 3 | 4 | 3 | 4 |
| 294 | 2 | 3 | 2 | 4 | 2 | 4 | 3 | 3 | 2 | 0 | 0 | 1 | 0 | 2 | 3 | 2 | 2 |
| 295 | 3 | 2 | 3 | 3 | 4 | 5 | 3 | 4 | 5 | 0 | 1 | 0 | 0 | 4 | 4 | 3 | 4 |
| 296 | 5 | 5 | 4 | 5 | 3 | 5 | 4 | 3 | 4 | 0 | 0 | 1 | 0 | 4 | 5 | 3 | 5 |
| 297 | 4 | 3 | 4 | 3 | 5 | 4 | 3 | 4 | 5 | 0 | 0 | 1 | 0 | 5 | 4 | 5 | 4 |
| 298 | 1 | 2 | 3 | 1 | 2 | 1 | 2 | 2 | 1 | 0 | 0 | 1 | 0 | 3 | 2 | 3 | 1 |
| 299 | 3 | 2 | 1 | 2 | 1 | 1 | 1 | 2 | 1 | 0 | 1 | 0 | 0 | 2 | 1 | 1 | 2 |
| 300 | 5 | 5 | 4 | 5 | 3 | 4 | 5 | 5 | 3 | 1 | 0 | 1 | 0 | 4 | 5 | 3 | 4 |
| 301 | 5 | 3 | 4 | 6 | 3 | 5 | 4 | 5 | 3 | 0 | 0 | 1 | 0 | 5 | 3 | 5 | 4 |
| 302 | 1 | 2 | 2 | 3 | 2 | 1 | 2 | 1 | 1 | 0 | 0 | 1 | 0 | 3 | 2 | 1 | 2 |
| 303 | 3 | 4 | 2 | 3 | 5 | 4 | 3 | 3 | 3 | 0 | 0 | 0 | 0 | 4 | 3 | 4 | 5 |
| 304 | 4 | 5 | 4 | 4 | 3 | 5 | 4 | 3 | 5 | 0 | 0 | 1 | 0 | 3 | 5 | 5 | 4 |
| 305 | 5 | 5 | 6 | 4 | 5 | 4 | 3 | 5 | 3 | 1 | 0 | 1 | 0 | 4 | 5 | 4 | 5 |
| 306 | 3 | 2 | 4 | 2 | 3 | 2 | 5 | 3 | 2 | 0 | 0 | 1 | 0 | 3 | 2 | 2 | 3 |
| 307 | 4 | 5 | 4 | 5 | 4 | 3 | 3 | 3 | 5 | 0 | 0 | 1 | 0 | 4 | 3 | 5 | 4 |
| 308 | 5 | 7 | 5 | 6 | 3 | 4 | 5 | 4 | 5 | 0 | 1 | 0 | 0 | 5 | 7 | 5 | 7 |
| 309 | 3 | 5 | 5 | 4 | 3 | 4 | 5 | 4 | 3 | 0 | 0 | 1 | 0 | 5 | 3 | 4 | 5 |
| 310 | 5 | 6 | 4 | 5 | 3 | 4 | 5 | 5 | 3 | 1 | 0 | 1 | 0 | 4 | 5 | 3 | 4 |
| 311 | 5 | 5 | 4 | 3 | 3 | 5 | 4 | 5 | 3 | 0 | 0 | 1 | 0 | 5 | 3 | 5 | 4 |
| 312 | 1 | 2 | 1 | 3 | 2 | 1 | 2 | 1 | 1 | 0 | 0 | 0 | 1 | 3 | 2 | 1 | 2 |
| 313 | 3 | 4 | 2 | 3 | 5 | 4 | 3 | 3 | 3 | 1 | 0 | 0 | 0 | 4 | 3 | 4 | 5 |
| 314 | 4 | 5 | 3 | 4 | 3 | 5 | 4 | 3 | 5 | 1 | 0 | 1 | 0 | 3 | 5 | 5 | 4 |
| 315 | 3 | 2 | 3 | 4 | 3 | 2 | 4 | 3 | 5 | 0 | 0 | 1 | 0 | 3 | 4 | 3 | 3 |
| 316 | 7 | 6 | 7 | 7 | 5 | 7 | 6 | 7 | 4 | 0 | 1 | 1 | 0 | 5 | 7 | 6 | 5 |
| 317 | 5 | 5 | 4 | 5 | 6 | 4 | 3 | 5 | 4 | 0 | 1 | 0 | 0 | 4 | 3 | 5 | 4 |
| 318 | 5 | 4 | 5 | 3 | 4 | 4 | 3 | 5 | 5 | 0 | 0 | 1 | 0 | 5 | 4 | 4 | 5 |
| 319 | 4 | 5 | 5 | 4 | 5 | 3 | 5 | 4 | 3 | 1 | 0 | 1 | 0 | 5 | 4 | 5 | 4 |
| 320 | 4 | 5 | 4 | 3 | 4 | 5 | 4 | 3 | 5 | 0 | 0 | 0 | 1 | 3 | 5 | 4 | 3 |
| 321 | 3 | 5 | 4 | 4 | 3 | 5 | 4 | 3 | 3 | 0 | 0 | 1 | 0 | 3 | 5 | 4 | 4 |
| 322 | 6 | 7 | 6 | 5 | 6 | 4 | 7 | 5 | 6 | 1 | 0 | 0 | 0 | 5 | 6 | 7 | 6 |
| 323 | 5 | 6 | 4 | 5 | 5 | 5 | 3 | 4 | 5 | 1 | 0 | 1 | 0 | 3 | 5 | 4 | 4 |
| 324 | 7 | 5 | 6 | 4 | 5 | 7 | 6 | 5 | 7 | 0 | 0 | 1 | 0 | 7 | 6 | 5 | 7 |
| 325 | 4 | 5 | 4 | 5 | 3 | 4 | 5 | 3 | 4 | 0 | 0 | 1 | 0 | 4 | 3 | 4 | 4 |
| 326 | 4 | 5 | 3 | 4 | 4 | 5 | 3 | 4 | 3 | 0 | 0 | 1 | 0 | 5 | 3 | 4 | 3 |
| 327 | 3 | 4 | 4 | 4 | 5 | 3 | 4 | 5 | 4 | 1 | 0 | 0 | 1 | 3 | 4 | 3 | 4 |
| 328 | 2 | 3 | 2 | 4 | 2 | 3 | 3 | 3 | 3 | 0 | 0 | 0 | 0 | 2 | 3 | 2 | 2 |
| 329 | 5 | 7 | 5 | 6 | 3 | 4 | 5 | 4 | 7 | 0 | 0 | 1 | 0 | 5 | 7 | 5 | 7 |
| 330 | 3 | 5 | 5 | 4 | 3 | 4 | 5 | 4 | 3 | 0 | 1 | 1 | 0 | 5 | 3 | 4 | 5 |
| 331 | 6 | 5 | 7 | 6 | 7 | 5 | 4 | 5 | 7 | 0 | 0 | 1 | 0 | 7 | 5 | 6 | 5 |
| 332 | 1 | 2 | 2 | 1 | 2 | 2 | 2 | 3 | 3 | 0 | 0 | 1 | 0 | 3 | 2 | 3 | 2 |
| 333 | 4 | 3 | 2 | 4 | 3 | 3 | 4 | 5 | 3 | 0 | 0 | 1 | 0 | 3 | 4 | 5 | 4 |
| 334 | 3 | 2 | 5 | 4 | 3 | 5 | 4 | 3 | 4 | 1 | 0 | 0 | 0 | 4 | 5 | 3 | 5 |
| 335 | 5 | 3 | 4 | 5 | 4 | 5 | 3 | 4 | 4 | 0 | 0 | 1 | 0 | 3 | 5 | 3 | 5 |
| 336 | 2 | 2 | 1 | 2 | 1 | 2 | 3 | 2 | 1 | 0 | 1 | 0 | 0 | 1 | 2 | 3 | 3 |
| 337 | 3 | 4 | 3 | 4 | 3 | 4 | 3 | 4 | 3 | 0 | 0 | 1 | 0 | 4 | 3 | 3 | 3 |
| 338 | 5 | 5 | 4 | 3 | 6 | 5 | 4 | 3 | 5 | 1 | 0 | 1 | 0 | 3 | 4 | 5 | 4 |
| 339 | 4 | 4 | 3 | 5 | 3 | 4 | 3 | 4 | 5 | 0 | 0 | 1 | 0 | 4 | 5 | 4 | 3 |
| 340 | 5 | 4 | 3 | 4 | 4 | 3 | 4 | 3 | 5 | 1 | 0 | 0 | 0 | 4 | 5 | 3 | 4 |
| 341 | 1 | 1 | 2 | 1 | 2 | 1 | 2 | 2 | 1 | 1 | 0 | 1 | 0 | 2 | 1 | 1 | 2 |
| 342 | 4 | 5 | 4 | 5 | 3 | 4 | 5 | 3 | 5 | 0 | 1 | 1 | 0 | 3 | 4 | 5 | 3 |
| 343 | 5 | 6 | 5 | 5 | 5 | 4 | 3 | 4 | 5 | 0 | 0 | 1 | 0 | 4 | 4 | 5 | 4 |
| 344 | 4 | 3 | 5 | 4 | 3 | 5 | 3 | 4 | 3 | 0 | 0 | 1 | 0 | 5 | 3 | 3 | 4 |
| 345 | 5 | 5 | 6 | 4 | 5 | 4 | 3 | 5 | 4 | 0 | 0 | 1 | 0 | 3 | 5 | 4 | 5 |
| 346 | 4 | 5 | 4 | 5 | 3 | 2 | 5 | 4 | 2 | 0 | 0 | 0 | 1 | 3 | 3 | 4 | 3 |
| 347 | 4 | 5 | 4 | 5 | 4 | 3 | 3 | 3 | 5 | 0 | 0 | 0 | 0 | 4 | 3 | 5 | 4 |
| 348 | 5 | 7 | 5 | 6 | 3 | 4 | 5 | 4 | 5 | 1 | 0 | 1 | 0 | 5 | 7 | 5 | 7 |
| 349 | 3 | 5 | 5 | 4 | 3 | 4 | 3 | 4 | 3 | 0 | 0 | 1 | 0 | 5 | 3 | 4 | 5 |
| 350 | 6 | 5 | 5 | 6 | 7 | 5 | 4 | 5 | 7 | 1 | 0 | 1 | 0 | 7 | 5 | 6 | 5 |
| 351 | 3 | 4 | 5 | 4 | 3 | 4 | 5 | 4 | 3 | 0 | 0 | 1 | 0 | 5 | 3 | 4 | 5 |
| 352 | 5 | 5 | 7 | 6 | 7 | 5 | 4 | 5 | 7 | 0 | 0 | 1 | 0 | 7 | 5 | 6 | 5 |
| 353 | 1 | 2 | 2 | 2 | 1 | 1 | 1 | 2 | 1 | 0 | 1 | 0 | 0 | 2 | 1 | 1 | 2 |
| 354 | 5 | 5 | 4 | 5 | 3 | 4 | 3 | 5 | 3 | 0 | 0 | 1 | 0 | 4 | 5 | 3 | 4 |
| 355 | 5 | 5 | 4 | 5 | 3 | 5 | 4 | 5 | 3 | 1 | 0 | 1 | 0 | 5 | 3 | 5 | 4 |
| 356 | 1 | 1 | 1 | 3 | 2 | 1 | 2 | 1 | 1 | 0 | 0 | 1 | 0 | 3 | 2 | 1 | 2 |
| 357 | 3 | 4 | 2 | 3 | 5 | 4 | 3 | 3 | 3 | 0 | 0 | 1 | 0 | 4 | 3 | 4 | 5 |
| 358 | 4 | 5 | 3 | 5 | 4 | 5 | 5 | 4 | 3 | 0 | 1 | 0 | 0 | 4 | 5 | 6 | 4 |
| 359 | 5 | 4 | 4 | 5 | 4 | 5 | 3 | 4 | 5 | 0 | 0 | 0 | 0 | 4 | 5 | 3 | 5 |
| 360 | 2 | 1 | 2 | 1 | 1 | 2 | 1 | 3 | 1 | 0 | 0 | 1 | 0 | 1 | 1 | 2 | 2 |
| 361 | 3 | 4 | 5 | 3 | 4 | 4 | 3 | 4 | 5 | 1 | 0 | 1 | 0 | 5 | 3 | 4 | 5 |
| 362 | 2 | 3 | 4 | 3 | 2 | 3 | 3 | 3 | 4 | 0 | 0 | 1 | 0 | 3 | 4 | 2 | 3 |
| 363 | 3 | 3 | 5 | 4 | 3 | 2 | 4 | 4 | 3 | 0 | 1 | 1 | 0 | 4 | 3 | 4 | 5 |
| 364 | 6 | 5 | 5 | 4 | 7 | 6 | 7 | 5 | 6 | 0 | 0 | 0 | 0 | 7 | 6 | 5 | 4 |
| 365 | 4 | 3 | 4 | 5 | 3 | 4 | 5 | 7 | 6 | 0 | 0 | 1 | 0 | 5 | 4 | 7 | 6 |
| 366 | 1 | 1 | 2 | 3 | 2 | 1 | 2 | 1 | 1 | 0 | 1 | 1 | 0 | 2 | 1 | 1 | 2 |
| 367 | 4 | 3 | 4 | 5 | 3 | 4 | 5 | 3 | 5 | 0 | 0 | 1 | 0 | 3 | 4 | 5 | 3 |
| 368 | 5 | 5 | 6 | 4 | 3 | 4 | 3 | 5 | 3 | 0 | 0 | 0 | 1 | 3 | 5 | 4 | 5 |
| 369 | 3 | 5 | 4 | 1 | 3 | 2 | 5 | 1 | 2 | 0 | 0 | 1 | 0 | 3 | 2 | 2 | 3 |
| 370 | 4 | 5 | 4 | 5 | 4 | 3 | 3 | 3 | 5 | 0 | 0 | 1 | 0 | 4 | 3 | 5 | 4 |
| 371 | 7 | 6 | 5 | 7 | 6 | 5 | 5 | 4 | 5 | 0 | 0 | 0 | 0 | 7 | 5 | 6 | 7 |
| 372 | 3 | 5 | 4 | 3 | 5 | 5 | 3 | 4 | 3 | 0 | 1 | 1 | 0 | 3 | 4 | 5 | 4 |
| 373 | 4 | 5 | 4 | 5 | 3 | 4 | 5 | 5 | 4 | 0 | 0 | 1 | 0 | 3 | 4 | 3 | 5 |
| 374 | 5 | 5 | 4 | 5 | 3 | 4 | 5 | 5 | 3 | 0 | 0 | 0 | 0 | 4 | 5 | 3 | 4 |
| 375 | 5 | 5 | 4 | 6 | 3 | 5 | 4 | 5 | 3 | 0 | 0 | 1 | 0 | 5 | 3 | 5 | 4 |
| 376 | 7 | 5 | 6 | 4 | 4 | 7 | 6 | 5 | 7 | 0 | 0 | 1 | 0 | 7 | 6 | 5 | 7 |
| 377 | 4 | 5 | 4 | 5 | 3 | 4 | 5 | 3 | 4 | 1 | 0 | 0 | 1 | 4 | 3 | 4 | 4 |
| 378 | 5 | 5 | 4 | 5 | 5 | 3 | 5 | 4 | 3 | 0 | 0 | 0 | 0 | 4 | 5 | 4 | 3 |
| 379 | 4 | 4 | 5 | 4 | 3 | 5 | 4 | 3 | 4 | 0 | 0 | 1 | 0 | 5 | 4 | 3 | 4 |
| 380 | 2 | 2 | 3 | 1 | 2 | 1 | 2 | 1 | 1 | 0 | 1 | 1 | 0 | 3 | 2 | 3 | 1 |
| 381 | 1 | 2 | 2 | 2 | 1 | 1 | 1 | 2 | 1 | 0 | 0 | 1 | 0 | 2 | 1 | 1 | 2 |
| 382 | 4 | 5 | 4 | 4 | 5 | 3 | 4 | 5 | 4 | 0 | 0 | 1 | 0 | 3 | 5 | 4 | 3 |
| 383 | 3 | 4 | 5 | 3 | 4 | 5 | 3 | 4 | 5 | 0 | 0 | 0 | 0 | 5 | 5 | 4 | 3 |
| 384 | 5 | 5 | 6 | 5 | 4 | 3 | 4 | 5 | 4 | 0 | 0 | 0 | 0 | 3 | 5 | 4 | 4 |
| 385 | 6 | 5 | 6 | 5 | 5 | 4 | 4 | 5 | 4 | 0 | 0 | 1 | 0 | 5 | 6 | 5 | 5 |
| 386 | 3 | 4 | 3 | 3 | 3 | 4 | 5 | 4 | 3 | 0 | 0 | 1 | 0 | 3 | 4 | 4 | 3 |
| 387 | 2 | 3 | 2 | 4 | 2 | 3 | 3 | 3 | 2 | 0 | 0 | 1 | 0 | 2 | 3 | 2 | 2 |
| 388 | 3 | 3 | 4 | 3 | 4 | 5 | 3 | 4 | 5 | 0 | 0 | 1 | 0 | 4 | 4 | 3 | 4 |
| 389 | 5 | 5 | 4 | 5 | 3 | 5 | 4 | 3 | 4 | 1 | 0 | 1 | 0 | 4 | 5 | 3 | 5 |
| 390 | 4 | 3 | 4 | 3 | 5 | 4 | 3 | 4 | 5 | 0 | 1 | 1 | 0 | 5 | 4 | 5 | 4 |
| 391 | 5 | 7 | 5 | 6 | 5 | 4 | 5 | 4 | 7 | 0 | 0 | 0 | 0 | 5 | 7 | 5 | 7 |
| 392 | 3 | 3 | 4 | 3 | 5 | 3 | 5 | 4 | 5 | 0 | 0 | 1 | 0 | 5 | 4 | 3 | 4 |
| 393 | 5 | 4 | 6 | 5 | 3 | 4 | 5 | 5 | 5 | 0 | 0 | 1 | 0 | 5 | 4 | 5 | 5 |
| 394 | 2 | 2 | 2 | 1 | 1 | 2 | 1 | 2 | 1 | 0 | 0 | 0 | 1 | 2 | 2 | 1 | 2 |
| 395 | 5 | 6 | 5 | 3 | 4 | 5 | 4 | 5 | 7 | 0 | 0 | 1 | 0 | 3 | 5 | 4 | 5 |
| 396 | 4 | 3 | 5 | 4 | 3 | 5 | 5 | 4 | 3 | 0 | 0 | 1 | 0 | 3 | 5 | 3 | 4 |
| 397 | 4 | 5 | 5 | 4 | 4 | 3 | 5 | 3 | 5 | 0 | 0 | 1 | 0 | 5 | 3 | 4 | 5 |
| 398 | 5 | 5 | 5 | 4 | 5 | 3 | 4 | 5 | 4 | 0 | 0 | 0 | 0 | 4 | 4 | 5 | 4 |
| 399 | 3 | 1 | 3 | 1 | 2 | 1 | 2 | 1 | 2 | 0 | 1 | 1 | 0 | 1 | 2 | 3 | 2 |
| 400 | 4 | 2 | 5 | 4 | 5 | 3 | 3 | 5 | 4 | 0 | 0 | 0 | 1 | 5 | 3 | 5 | 4 |
| 401 | 3 | 2 | 3 | 4 | 3 | 2 | 4 | 3 | 3 | 0 | 0 | 1 | 0 | 3 | 4 | 3 | 3 |
| 402 | 7 | 6 | 7 | 7 | 5 | 7 | 6 | 7 | 7 | 0 | 0 | 0 | 0 | 5 | 7 | 6 | 5 |
| 403 | 5 | 5 | 4 | 5 | 6 | 4 | 3 | 5 | 4 | 1 | 0 | 1 | 0 | 4 | 3 | 5 | 4 |
| 404 | 4 | 4 | 5 | 3 | 3 | 3 | 3 | 5 | 5 | 1 | 0 | 1 | 0 | 5 | 4 | 4 | 5 |
| 405 | 4 | 3 | 5 | 4 | 5 | 3 | 5 | 4 | 3 | 0 | 0 | 1 | 0 | 5 | 4 | 5 | 4 |
| 406 | 4 | 3 | 5 | 4 | 3 | 3 | 4 | 5 | 3 | 0 | 0 | 1 | 0 | 4 | 3 | 4 | 3 |
| 407 | 3 | 4 | 5 | 3 | 4 | 3 | 3 | 4 | 5 | 0 | 0 | 0 | 0 | 5 | 4 | 3 | 4 |
| 408 | 5 | 6 | 4 | 4 | 3 | 5 | 4 | 3 | 4 | 0 | 0 | 1 | 0 | 5 | 4 | 5 | 5 |
| 409 | 6 | 5 | 7 | 7 | 7 | 5 | 6 | 7 | 6 | 0 | 1 | 1 | 0 | 5 | 6 | 7 | 7 |
| 410 | 3 | 3 | 5 | 4 | 3 | 2 | 5 | 3 | 4 | 0 | 0 | 0 | 0 | 4 | 3 | 4 | 5 |
| 411 | 2 | 2 | 3 | 3 | 2 | 3 | 2 | 3 | 4 | 0 | 0 | 1 | 0 | 2 | 2 | 2 | 3 |
| 412 | 5 | 6 | 5 | 6 | 7 | 5 | 6 | 7 | 4 | 0 | 0 | 1 | 0 | 6 | 7 | 7 | 7 |
| 413 | 3 | 5 | 3 | 5 | 4 | 4 | 5 | 3 | 4 | 0 | 0 | 1 | 0 | 3 | 5 | 4 | 4 |
| 414 | 2 | 2 | 3 | 4 | 3 | 4 | 2 | 3 | 4 | 0 | 0 | 1 | 0 | 3 | 2 | 3 | 3 |
| 415 | 5 | 3 | 5 | 4 | 3 | 5 | 4 | 3 | 4 | 0 | 1 | 1 | 0 | 4 | 3 | 5 | 4 |
| 416 | 4 | 1 | 3 | 4 | 3 | 4 | 1 | 1 | 1 | 0 | 0 | 1 | 0 | 3 | 1 | 1 | 2 |
| 417 | 6 | 6 | 7 | 7 | 5 | 6 | 6 | 7 | 7 | 0 | 0 | 0 | 0 | 5 | 6 | 7 | 7 |
| 418 | 5 | 6 | 5 | 4 | 4 | 5 | 4 | 5 | 3 | 1 | 0 | 1 | 0 | 4 | 3 | 4 | 5 |
| 419 | 6 | 5 | 5 | 4 | 6 | 6 | 7 | 5 | 6 | 0 | 0 | 1 | 0 | 7 | 6 | 5 | 4 |
| 420 | 3 | 4 | 4 | 3 | 4 | 3 | 4 | 4 | 3 | 0 | 0 | 1 | 0 | 3 | 4 | 5 | 3 |
| 421 | 7 | 6 | 5 | 7 | 6 | 7 | 5 | 4 | 5 | 0 | 1 | 0 | 1 | 7 | 5 | 6 | 7 |
| 422 | 3 | 5 | 4 | 3 | 5 | 5 | 3 | 4 | 2 | 0 | 0 | 0 | 0 | 3 | 4 | 5 | 4 |
| 423 | 4 | 5 | 4 | 3 | 3 | 4 | 5 | 5 | 4 | 0 | 0 | 1 | 0 | 3 | 4 | 3 | 5 |
| 424 | 5 | 5 | 3 | 5 | 3 | 4 | 5 | 5 | 3 | 1 | 0 | 0 | 0 | 4 | 5 | 3 | 4 |
| 425 | 5 | 4 | 4 | 6 | 3 | 5 | 4 | 5 | 3 | 0 | 0 | 1 | 0 | 5 | 3 | 5 | 4 |
| 426 | 1 | 2 | 1 | 3 | 3 | 1 | 2 | 1 | 1 | 0 | 0 | 1 | 0 | 3 | 2 | 1 | 2 |
| 427 | 3 | 4 | 2 | 3 | 5 | 4 | 3 | 3 | 4 | 0 | 0 | 0 | 1 | 4 | 3 | 4 | 5 |
| 428 | 4 | 5 | 3 | 4 | 3 | 5 | 6 | 3 | 5 | 0 | 1 | 1 | 0 | 3 | 5 | 5 | 4 |
