# Supplementary material for: Research on the path of green technology innovation driven by the Environmental Protection Tax Law: Based on data of heavy polluting enterprises
Source: PLoS One. 2024 Jul 30;19(7):e0308215. doi: 10.1371/journal.pone.0308215 (PMC11288438; doi:10.1371/journal.pone.0308215)
Supplement: Attachment — Submitted filename: Response to Reviewers.docx [file pone.0308215.s002.docx]

Response to Reviewers

Research on the green technology innovation-driven path regulated by the Environmental Protection Tax Law in the post-epidemic era

Dear Editor：

We would like to thank the editor and all the reviewers for the constructive feedback and suggestions concerning the previous version of the manuscript. We are very pleased to have had the opportunity to revise and resubmit this improved version of our manuscript.

In the following, we offer detailed responses to the Reviewers’ comments.

Yours faithfully

*The Authors*

# Additional Editor Comments (REV_A) Highlight in red.

| **Co mm ent**  **No.** | **Page No.** | **Reviewer #A: Comments** | **Amendments** |
| --- | --- | --- | --- |
| REV_  A_1 |  | the Introduction and Literature Review sections do not do a good job of highlighting the marginal contributions of this paper and need to be reworked. | Thank you for your valuable comments. The author has revised the introduction and literature review. The details are as follows:  1. Introduction  Due to the rapid economic growth, environmental problems have become a key factor restricting the healthy development of China's economy [1]. The relationship between economic growth and environmental protection is not difficult to reconcile, on the contrary, with the help of necessary environmental policy tools can achieve economic and environmental win-win results [2]. Environmental regulation has become an important policy tool to alleviate environmental problems [3]. China also hopes to achieve the common development of the environment and economy with the help of environmental regulation policy tools. On January 1, 2018, the Chinese EPTL was formally implemented, a major event in the course of China's economic and social development, not only marking the formal establishment of China's environmental protection tax system but also demonstrating the Chinese government's attitude and determination to make economic growth and environmental protection a common development objective [4]. The implementation of EPTL in developed countries has a history of more than a hundred years, and rich skills and experience have been accumulated, which has played a positive role in economic growth and environmental protection [5]. However, the normal implementation process of the EPTL in our country has suffered from an abnormal impact and produced the dissimilation effect. In the new economic situation, it is necessary to deeply analyze the dissimilation effect of the EPTL, as well as to propose new corrective and optimization measures to promote the EPTL to play its due value. Thus driving the realization of green technology innovation and effective use of environmental resources [6]. The realization of green technology innovation is of great significance to green economic development and the correction of environmental behavior [7], while the Porter hypothesis effect of EPTL stimulates green technology innovation [8].  The implementation of China’s EPTL has been incubated and explored for more than ten years. In May 2007, China’s State Council issued the Comprehensive Work Program for Energy Conservation and Emission Reduction, which began to explore the issue of environmental taxation. In June 2007, China’s Ministry of Finance (MOF), the General Administration of Taxation (GAT), and the Ministry of Environmental Protection (MEP) set up a Joint Study Group to discuss several issues in the introduction of environmental tax and submitted the draft environmental tax to the State Council at the end of 2014. Subsequently, the Joint Study Group went out for public comment on two more occasions in 2015 and 2016. On December 25, 2016, the Standing Committee of the National People's Congress (NPC) considered and passed the EPTL. On July 21, 2017, China’s MOF, GAT, and MEP jointly promulgated the Notice on Comprehensively Preparing for the Implementation of the EPTL. On December 22, 2017, the State Council issued a Notice on the Attribution of Environmental Protection Tax Revenue. On December 25, 2017, the State Council promulgated the Regulations on the Implementation of the EPTL. On December 27, 2017, the MEP issued the Announcement on the Release of Pollution Coefficients and Material Accounting Methods for Calculating Pollutant Emissions. On January 1, 2018, the EPTL came into effect. On January 7, 2018, the MOF issued the Notice on Matters Related to the Suspension of Sewage Charges and Other Administrative Fees. As an important part of the world, China's environmental behavior will be conducive to the improvement of the global environment. China's EPTL is a mandatory environmental governance strategy led by the government [9], and the key is to achieve a win-win situation between the government's environmental concern and corporate financial performance.  As a typical environmental regulation, the EPTL also has the characteristics of the Porter hypothesis effect [10]. Porter (1991) states that under the right market environment, stringent environmental regulations can incentivize firms to implement in-depth innovation and enhance green technological innovation capabilities, partially or completely offsetting the increase in firms' costs under environmental regulations, thus giving firms a competitive advantage in the marketplace, which is the core idea of the Porter hypothesis [11]. Jaffe and Palmer (1997) based on a large number of enterprise environmental regulation case analyses, and further analysis of the Porter hypothesis, argue that if the enterprise's green technology innovation partially offsets the cost increase under environmental regulation, it is called the Weak Porter hypothesis. If enterprises' green technology innovation completely offsets the cost increase under environmental regulation, it is called the Strong Porter hypothesis [12]. It can be seen that the transition from the Weak Porter hypothesis to the Strong Porter hypothesis is the process of gradually realizing the function of environmental regulation. At the same time, more resources can be allocated to sustainable and environmentally friendly projects, to achieve green development [13]. Therefore, environmental regulations will attract more market participants into the green technology market and achieve green innovation [14].  In environmental economic theory, the Porter hypothesis effect is an effective way to test the effectiveness of the implementation of the EPTL. A few studies show that the Porter hypothesis effect of China’s EPTL has not yet been realized. Based on the sample data of A-share listed companies in Shanghai and Shenzhen from 2011 to 2019, Yuan (2022) utilized the double-difference method and found that the implementation of the EPTL significantly inhibited the green technological innovation capability of enterprises, especially more profoundly and extensively inhibited the green technological innovation capability of state-owned enterprises [15]. The gap between the implementation of environmental laws and regulations and the original intention of legislation makes China's EPTL seriously ineffective [16], which is not conducive to the mitigation of environmental problems, but also hurts the innovation ability of enterprises. In the face of strict EPTL, enterprises will adopt greenwashing behavior to avoid punishment, which is not only detrimental to the shaping of green technology innovation ability but also brings serious harm to the whole society [17].  However, most studies show that the Porter hypothesis effect of China's EPTL has already appeared, but is in an imperfect state. Wen and Zhong (2020) found that the environmental protection tax reform has a significant Porter hypothesis effect on large and medium-sized enterprises, which significantly promotes the growth of green technology innovation in large and medium-sized enterprises, but has no significant impact on small enterprises. Therefore, it is suggested to implement supporting policies to reduce the adverse impact of environmental protection tax on small and micro enterprises [18]. Cui, Lu, and Wang (2021) took China's A-share listed heavy polluting enterprises from 2014 to 2019 as samples and found that the implementation of the Environmental protection tax significantly increased the investment of China's heavy polluting enterprises and enhanced their green technology innovation, and there was still a large space for the promotion of green technology innovation [19]. Leng and Quan (2021) argued that as an environmental regulation, the EPTL has a natural driving effect on green innovation, but at present, the EPTL only has a driving effect on green innovation in the process of production and operation of enterprises, and it can't yet have a driving effect on the green innovation in the result of production and operation [20]. The focus of environmental protection tax also affects the effect of green technology innovation, but the government paying more attention to environmental issues will hurt the finance of enterprises, and thus endanger the investment in technological innovation [21]. Frequent changes in economic policies have inhibited green technology innovation [22], making it difficult to achieve the goal of environmental protection tax. In the study of environmental regulation policy, some scholars believe that relying solely on the mandatory environmental protection tax is not conducive to the green technology innovation ability [23][24][25], which makes it difficult to fully realize the Porte hypothesis effect of EPTL.  Some studies show that China's EPTL not only has the Porter hypothesis effect but also the effect of regional differences on the Porter hypothesis can not be ignored. Hu, Chen, and Zhao (2020) tested the panel data of high-polluting manufacturing enterprises in 30 provincial regions in China from 2008 to 2018 and found that environmental protection tax has a significant promoting effect on green technology innovation of high-polluting enterprises, but the Porter hypothesis effect of environmental protection tax lagging one period is more obvious, and pointed out that regional differences are an important factor affecting the effect of Porter hypothesis of environmental protection tax [26]. Also taking Chinese listed companies from 2010 to 2018 as research objects, Kong et al. (2024) believe that in addition to geographical differences, differences in corporate ownership structure will also have an impact on the effect of environmental protection tax [27].  Existing research shows that China's EPTL has shown the Porter hypothesis effect, which to a certain extent has promoted the green technological innovation of enterprises. However, with the advancement of the process, the promoting effect of green technology innovation is declining. Existing studies have rarely mentioned the alienating effect of environmental protection tax in China, nor have they discussed the internal mechanism in-depth, nor have they conducted a comprehensive review of the promoting effect of environmental protection tax on the technological innovation ability of enterprises. As a compulsory environmental regulation, the environmental regulation behavior and the environmental regulation effect of environmental protection tax have not been fully discussed. Therefore, to implement the EPTL and get rid of the framework of dissimilation effect as soon as possible, it is necessary to deeply analyze the dissimilation mechanism of the environmental protection tax collection and management, and propose improvement plans to improve the quality of the environmental protection tax collection and management, to maximize the green technological innovation and corporate competitive advantage of enterprises.  The innovations of this paper can be summarized as follows: First, the impact of environmental regulations on green technology innovation is explored, revealing the role of government controls on firms' technological development. Second, the green technology innovation driving path based on China's environmental protection tax is explored through empirical tests. Thirdly, the paper analyzes the role of environmental regulation in China's social and economic background under the framework of the dissimilation effect and further verifies the role of the Porter hypothesis in China's environmental background by constructing a research model set of dissimilation effects. |
| REV_  A_2 |  | the model has a lot of independent variables and is very prone to the problem of multicollinearity, and the article lacks relevant tests. | Thank you very much for your questions. Before the study of model testing, multicollinearity tests were carried out, and both were described in the paper. For fear of too much space, the correlation table was not placed in the paper. |
| REV_  A_3 |  | only the correlation coefficient values are shown in Table 3, usually, the t-value statistic or standard deviation of the coefficient values are also needed to be shown, which is recommended to be added.Other forms have the same problem. | Thank you very much for your questions. After the relative value, the p-value is indicated, which also indicates significance and has the same effect as the T-value. The details are as follows: Table 4 Test results of the research model \| Green process innovation ($Gcra$) \| Green product innovation ($Gcom$) \| End treatment innovation ($Gend$) \| Green technology innovation ($Gtec$); Coordination between levies and administrations ($xt)$ \| 0.05(0.0632) \| 0.03(0.0702) \| 0.12**(0.0072) \| 0.07(0.0611); Emissions monitoring science ($jc)$ \| 0.12**(0.0063) \| 0.13***(0.0005) \| 0.15***(0.0008) \| 0.14***(0.0003); Differential tax rate setting $(cy)$ \| 0.09*(0.0315) \| 0.07(0.0858) \| 0.11**(0.0076) \| 0.10*(0.0332); Tax information sharing $(gx)$ \| 0.02(0.1121) \| 0.01(0.0871) \| 0.03(0.0772) \| 0.00(0.0518); Definition of the scope of levy and administration ($fw)$ \| 0.08*(0.0405) \| 0.10**(0.0059) \| 0.07(0.0606) \| 0.09*(0.0323); Tax declaration counseling $(fd)$ \| 0.07(0.0922) \| 0.05(0.0688) \| 0.09*(0.0303) \| 0.03(0.0627); Accountability mechanism design ($wz)$ \| 0.11**(0.0059) \| 0.09*(0.0401) \| 0.13***(0.0005) \| 0.10**(0.0027); Implementation of preferential policies ($yh)$ \| 0.13***(0.0006) \| 0.08*(0.0328) \| 0.15***(0.0008) \| 0.12***(0.0006); Tax rate level verification $(sl)$ \| 0.02(0.1031) \| 0.03(0.0962) \| 0.10*(0.0356) \| 0.06(0.0708); Medium-sized enterprise $(me)$ \| 0.12**(0.0059) \| 0.07(0.0602) \| 0.14***(0.0002) \| 0.13***(0.0004); Large-sized enterprise $(be)$ \| 0.10*(0.0202) \| 0.05(0.0662) \| 0.11***(0.0007) \| 0.09*(0.0215); $R^{2}$ \| 0.69 \| 0.70 \| 0.62 \| 0.66; $\Delta R^{2}$ \| 0.01 \| 0.02 \| 0.02 \| 0.01; adjusted$R^{2}$ \| 0.70 \| 0.72 \| 0.64 \| 0.67; adjusted $F$Value \| 108.36 \| 88.19 \| 79.26 \| 138.76; $P$Value \| **(0.0033) \| ***(0.0004) \| **(0.0052) \| ***(0.0005) Notes: *P<0.05; **P<0.01; ***P<0.001; N=428; The values in parentheses are P value. Table 5 Test results of the research model (robustness test) \| Green process innovation ($Gcra$) \| Green product innovation ($Gcom$) \| End treatment innovation ($Gend$) \| Green technology innovation ($Gtec$); Coordination between levies and administrations ($xt)$ \| 0.07(0.1032) \| 0.05(0.1164) \| 0.12**(0.0062) \| 0.08(0.0645); Emissions monitoring science ($jc)$ \| 0.12**(0.0084) \| 0.13***(0.0007) \| 0.12**(0.0067) \| 0.11**(0.0052); Differential tax rate setting $(cy)$ \| 0.10*(0.0427) \| 0.03(0.0976) \| 0.13**(0.0084) \| 0.10*(0.0382); Tax information sharing $(gx)$ \| 0.05(0.1028) \| 0.01(0.0981) \| 0.05(0.0655) \| 0.03(0.0852); Definition of the scope of levy and administration ($fw)$ \| 0.11*(0.0452) \| 0.13**(0.0086) \| 0.07(0.0583) \| 0.13*(0.0355); Tax declaration counseling $(fd)$ \| 0.07(0.1076) \| 0.05(0.0927) \| 0.11*(0.0337) \| 0.05(0.0876); Accountability mechanism design ($wz)$ \| 0.11**(0.0052) \| 0.12*(0.0257) \| 0.13**(0.0076) \| 0.11*(0.0411); Implementation of preferential policies ($yh)$ \| 0.10**(0.0064) \| 0.11*(0.0322) \| 0.12**(0.0083) \| 0.14***(0.0007); Tax rate level verification $(sl)$ \| 0.02(0.0623) \| 0.05(0.0877) \| 0.10*(0.0201) \| 0.06(0.0768); Medium-sized enterprise $(me)$ \| 0.15***(0.0004) \| 0.07(0.0966) \| 0.13**(0.0064) \| 0.15***(0.0006); Large-sized enterprise $(be)$ \| 0.14**(0.0065) \| 0.02(0.0662) \| 0.12**(0.0072) \| 0.13*(0.0352); Private enterprise $(pe)$ \| -0.13***(0.0005) \| -0.11**(0.0056) \| -0.12**(0.0084) \| -0.14***(0.0006); Foreign-funded enterprise $(fe)$ \| 0.10*(0.0266) \| 0.11*(0.0314) \| 0.12**(0.0056) \| 0.16***(0.0004); Central China ($mi)$ \| -0.08(0.0866) \| -0.10*(0.0288) \| -0.02(0.0705) \| -0.12**(0.0064); Western China ($we)$ \| -0.11**(0.0047) \| -0.02(0.1035) \| -0.14***(0.0005) \| -0.02(0.0688); $R^{2}$ \| 0.72 \| 0.68 \| 0.66 \| 0.67; $\Delta R^{2}$ \| 0.05 \| 0.02 \| 0.02 \| 0.04; adjusted$R^{2}$ \| 0.77 \| 0.70 \| 0.68 \| 0.71; adjusted $F$Value \| 123.29 \| 92.78 \| 100.26 \| 87.19; $P$Value \| **(0.0067) \| **(0.0026) \| ***(0.0008) \| ***(0.0005) Notes: *P<0.05; **P<0.01; ***P<0.001; N=428; The values in parentheses are P value. |
| REV_  A_4 |  | the recommendations of the article are a bit long and it is recommended to analyse them in conjunction with the conclusions. | Thank you for your valuable comment. The author has rewritten the conclusions and countermeasures as follows:  *4.1* Conclusions  Through the comprehensive judgment of the test results, the following conclusions can be drawn. First, the environmental regulatory acts of the EPTL, such as the coordination of collection and management departments, the sharing of tax collection information, the counseling on tax declaration, and the approval of tax rate levels, have not produced a substantial effect on the promotion of enterprises' green technological innovation, while the rest of the regulatory acts have facilitated the growth of the enterprises' green technological innovation capacity to varying degrees. Second, the Porter hypothesis effect of the EPTL is most pronounced in medium-sized enterprises, weakest in large-sized enterprises, and second weakest in small enterprises. Third, the Porter hypothesis effect of the EPTL is the most obvious in foreign-funded enterprises, followed by state-owned enterprises, and the weakest in private enterprises.  In conclusion, with the implementation of the EPTL, regulatory behavior difficult to ensure the effectiveness of the EPTL. The main performance is Porter hypothesis effect has been significantly weakened, the driving force for enterprises' green technology innovation is decreased, and most regulatory behaviors hinder the occurrence of enterprises' green technology innovation. Therefore, this unfavorable situation should be reversed as soon as possible, the Porter hypothesis effect should be restored, and the environmental regulatory function of the EPTL should be fully realized, to vigorously enhance the green technological innovation capability of enterprises.  By constructing the dissimilarity effect research model group to empirically test the green technology innovation driving path of China's environmental protection tax, this study expands the application of the Porter hypothesis effect in China's management context, further reveals the micro-mechanism of the environmental regulatory effect of the EPTL, and provides theoretical references and practical bases for the realization of the co-development of the economy and the environment for the rest of the world countries and regions.  *4.2*Policy implications  According to the test results, combined with the investigation on the implementation of China's EPTL, it can be seen that to more effectively drive the growth of green technological innovation in enterprises, the reform and improvement of the EPTL should be focused on promoting in the following directions. First, strengthen the contact and communication between the tax department and the environmental protection department at the senior management. According to the empirical test results, the coordination between levies and administrations has not achieved the drive of green process innovation, green product innovation, and green technology innovation. Therefore, it is necessary to break the business gap between the tax department and the environmental protection department, improve the coordination in the operation process, and ensure the effect of the implementation of the EPTL. Second, local government departments need to vigorously promote the construction of information sharing platforms for environmental protection tax. According to the empirical test results, the regulatory behavior of tax information sharing does not play a driving role in the green technology innovation ability of enterprises. Local governments need to personally guide environmental protection agencies, tax agencies, and tax-paying enterprises to participate together, to vigorously promote the process of information sharing platform construction. Third, the tax authorities need to increase the business training of enterprise taxpayers. According to the empirical test results, the regulation behavior of tax declaration counseling has a serious alienation effect, which only plays a role in the end treatment innovation. Therefore, the tax authorities need to send experienced tax personnel to participate in the training work and change the training method to open the training situation as soon as possible. Finally, flexibly adjust the tax rate level of regional environmental protection tax. According to the empirical test results, the regulatory behavior of tax rate level verification is conducive to end-treatment innovation, but not conducive to green process innovation, green product innovation, and green technology innovation. Therefore, each region should establish the most effective tax rate according to their economy, society, and environmental protection, to promote the dual development of environmental protection and economic growth. |
| REV_  A_5 |  | the reviewer's recommended literature is optional, if it is not relevant, there is no need to force the citation of irrelevant literature. | Thank you for your valuable comment. The author makes reference to some relevant literatures. |

# Reviewer#1(REV_1)

| **Co mm ent**  **No.** | **Pa**  **ge**  **No**  **.** | **Reviewer 1: Comments** | **Amendments** |
| --- | --- | --- | --- |
| REV_  1_1 |  | Introduction of the paper is weak and does not have flow | Thank you for your valuable comment. The author has revised the introduction to achieve higher quality.  1. Introduction  Due to the rapid economic growth, environmental problems have become a key factor restricting the healthy development of China's economy [1]. The relationship between economic growth and environmental protection is not difficult to reconcile, on the contrary, with the help of necessary environmental policy tools can achieve economic and environmental win-win results [2]. Environmental regulation has become an important policy tool to alleviate environmental problems [3]. China also hopes to achieve the common development of the environment and economy with the help of environmental regulation policy tools. On January 1, 2018, the Chinese EPTL was formally implemented, a major event in the course of China's economic and social development, not only marking the formal establishment of China's environmental protection tax system but also demonstrating the Chinese government's attitude and determination to make economic growth and environmental protection a common development objective [4]. The implementation of EPTL in developed countries has a history of more than a hundred years, and rich skills and experience have been accumulated, which has played a positive role in economic growth and environmental protection [5]. However, the normal implementation process of the EPTL in our country has suffered from an abnormal impact and produced the dissimilation effect. In the new economic situation, it is necessary to deeply analyze the dissimilation effect of the EPTL, as well as to propose new corrective and optimization measures to promote the EPTL to play its due value. Thus driving the realization of green technology innovation and effective use of environmental resources [6]. The realization of green technology innovation is of great significance to green economic development and the correction of environmental behavior [7], while the Porter hypothesis effect of EPTL stimulates green technology innovation [8].  The implementation of China’s EPTL has been incubated and explored for more than ten years. In May 2007, China’s State Council issued the Comprehensive Work Program for Energy Conservation and Emission Reduction, which began to explore the issue of environmental taxation. In June 2007, China’s Ministry of Finance (MOF), the General Administration of Taxation (GAT), and the Ministry of Environmental Protection (MEP) set up a Joint Study Group to discuss several issues in the introduction of environmental tax and submitted the draft environmental tax to the State Council at the end of 2014. Subsequently, the Joint Study Group went out for public comment on two more occasions in 2015 and 2016. On December 25, 2016, the Standing Committee of the National People's Congress (NPC) considered and passed the EPTL. On July 21, 2017, China’s MOF, GAT, and MEP jointly promulgated the Notice on Comprehensively Preparing for the Implementation of the EPTL. On December 22, 2017, the State Council issued a Notice on the Attribution of Environmental Protection Tax Revenue. On December 25, 2017, the State Council promulgated the Regulations on the Implementation of the EPTL. On December 27, 2017, the MEP issued the Announcement on the Release of Pollution Coefficients and Material Accounting Methods for Calculating Pollutant Emissions. On January 1, 2018, the EPTL came into effect. On January 7, 2018, the MOF issued the Notice on Matters Related to the Suspension of Sewage Charges and Other Administrative Fees. As an important part of the world, China's environmental behavior will be conducive to the improvement of the global environment. China's EPTL is a mandatory environmental governance strategy led by the government [9], and the key is to achieve a win-win situation between the government's environmental concern and corporate financial performance.  As a typical environmental regulation, the EPTL also has the characteristics of the Porter hypothesis effect [10]. Porter (1991) states that under the right market environment, stringent environmental regulations can incentivize firms to implement in-depth innovation and enhance green technological innovation capabilities, partially or completely offsetting the increase in firms' costs under environmental regulations, thus giving firms a competitive advantage in the marketplace, which is the core idea of the Porter hypothesis [11]. Jaffe and Palmer (1997) based on a large number of enterprise environmental regulation case analyses, and further analysis of the Porter hypothesis, argue that if the enterprise's green technology innovation partially offsets the cost increase under environmental regulation, it is called the Weak Porter hypothesis. If enterprises' green technology innovation completely offsets the cost increase under environmental regulation, it is called the Strong Porter hypothesis [12]. It can be seen that the transition from the Weak Porter hypothesis to the Strong Porter hypothesis is the process of gradually realizing the function of environmental regulation. At the same time, more resources can be allocated to sustainable and environmentally friendly projects, to achieve green development [13]. Therefore, environmental regulations will attract more market participants into the green technology market and achieve green innovation [14].  In environmental economic theory, the Porter hypothesis effect is an effective way to test the effectiveness of the implementation of the EPTL. A few studies show that the Porter hypothesis effect of China’s EPTL has not yet been realized. Based on the sample data of A-share listed companies in Shanghai and Shenzhen from 2011 to 2019, Yuan (2022) utilized the double-difference method and found that the implementation of the EPTL significantly inhibited the green technological innovation capability of enterprises, especially more profoundly and extensively inhibited the green technological innovation capability of state-owned enterprises [15]. The gap between the implementation of environmental laws and regulations and the original intention of legislation makes China's EPTL seriously ineffective [16], which is not conducive to the mitigation of environmental problems, but also hurts the innovation ability of enterprises. In the face of strict EPTL, enterprises will adopt greenwashing behavior to avoid punishment, which is not only detrimental to the shaping of green technology innovation ability but also brings serious harm to the whole society [17].  However, most studies show that the Porter hypothesis effect of China's EPTL has already appeared, but is in an imperfect state. Wen and Zhong (2020) found that the environmental protection tax reform has a significant Porter hypothesis effect on large and medium-sized enterprises, which significantly promotes the growth of green technology innovation in large and medium-sized enterprises, but has no significant impact on small enterprises. Therefore, it is suggested to implement supporting policies to reduce the adverse impact of environmental protection tax on small and micro enterprises [18]. Cui, Lu, and Wang (2021) took China's A-share listed heavy polluting enterprises from 2014 to 2019 as samples and found that the implementation of the Environmental protection tax significantly increased the investment of China's heavy polluting enterprises and enhanced their green technology innovation, and there was still a large space for the promotion of green technology innovation [19]. Leng and Quan (2021) argued that as an environmental regulation, the EPTL has a natural driving effect on green innovation, but at present, the EPTL only has a driving effect on green innovation in the process of production and operation of enterprises, and it can't yet have a driving effect on the green innovation in the result of production and operation [20]. The focus of environmental protection tax also affects the effect of green technology innovation, but the government paying more attention to environmental issues will hurt the finance of enterprises, and thus endanger the investment in technological innovation [21]. Frequent changes in economic policies have inhibited green technology innovation [22], making it difficult to achieve the goal of environmental protection tax. In the study of environmental regulation policy, some scholars believe that relying solely on the mandatory environmental protection tax is not conducive to the green technology innovation ability [23][24][25], which makes it difficult to fully realize the Porte hypothesis effect of EPTL.  Some studies show that China's EPTL not only has the Porter hypothesis effect but also the effect of regional differences on the Porter hypothesis can not be ignored. Hu, Chen, and Zhao (2020) tested the panel data of high-polluting manufacturing enterprises in 30 provincial regions in China from 2008 to 2018 and found that environmental protection tax has a significant promoting effect on green technology innovation of high-polluting enterprises, but the Porter hypothesis effect of environmental protection tax lagging one period is more obvious, and pointed out that regional differences are an important factor affecting the effect of Porter hypothesis of environmental protection tax [26]. Also taking Chinese listed companies from 2010 to 2018 as research objects, Kong et al. (2024) believe that in addition to geographical differences, differences in corporate ownership structure will also have an impact on the effect of environmental protection tax [27].  Existing research shows that China's EPTL has shown the Porter hypothesis effect, which to a certain extent has promoted the green technological innovation of enterprises. However, with the advancement of the process, the promoting effect of green technology innovation is declining. Existing studies have rarely mentioned the alienating effect of environmental protection tax in China, nor have they discussed the internal mechanism in-depth, nor have they conducted a comprehensive review of the promoting effect of environmental protection tax on the technological innovation ability of enterprises. As a compulsory environmental regulation, the environmental regulation behavior and the environmental regulation effect of environmental protection tax have not been fully discussed. Therefore, to implement the EPTL and get rid of the framework of dissimilation effect as soon as possible, it is necessary to deeply analyze the dissimilation mechanism of the environmental protection tax collection and management, and propose improvement plans to improve the quality of the environmental protection tax collection and management, to maximize the green technological innovation and corporate competitive advantage of enterprises.  The innovations of this paper can be summarized as follows: First, the impact of environmental regulations on green technology innovation is explored, revealing the role of government controls on firms' technological development. Second, the green technology innovation driving path based on China's environmental protection tax is explored through empirical tests. Thirdly, the paper analyzes the role of environmental regulation in China's social and economic background under the framework of the dissimilation effect and further verifies the role of the Porter hypothesis in China's environmental background by constructing a research model set of dissimilation effects. |
| REV_  1_2 |  | Abstract needs to rewrite | Thank you for your helpful suggestions. The author has rewritten the abstract.  **Abstract:** Environmental Protection Tax Law (EPTL) is a compulsory environmental regulation measure adopted by China to deal with environmental problems. However, with the advancement of implementation, the EPTL produces a dissimilation effect and damages the realization of the Porter hypothesis effect. The study examines the dissimilation effect of green technology innovation regulated by the EPTL using sample data from heavy pollution firms in China. According to the empirical test results: (1) the coordination between levies and administrations, differential tax rate setting, tax information sharing, definition of the scope of levy and administration, tax declaration counseling, and tax rate level verification produce the dissimilation effect; (2) the Porter hypothesis effect of the EPTL is the most significant in medium-sized enterprises and foreign-funded enterprises. By constructing the research model group of dissimilation effect, this study analyzes the application of environmental regulation in China's social and economic background, thus providing a reference for developing of the green economy. |
| REV_  1_3 |  | Discussion part is required to rewrite, author is expected to discuss the inferences of analysis | Thank you for your valuable advice. The author reorganizes the discussion part and analyzes the economic significance behind it.  *3.3* Discussion  The study empirically tests the effects of the Porter hypothesis of the EPTL by constructing a research model set of the dissimilation effects of green technological innovations regulated by the EPTL and utilizing sample data from heavy polluting enterprises in China. According to the results of the model test, it can be seen that with the implementation of China's EPTL, the promotion effect of environmental regulation on green technological innovation has been alienated, and the effect of Porter hypothesis has failed to be fully realized, and the realization of the green technological innovation ability of heavy polluting enterprises has been impeded by the existing regulatory behavior.  he environmental regulation effect of the EPTL has been proved [42], and the effective implementation of the tax law can urge enterprises to take positive measures to deal with environmental punishment, to increase their investment in green technology innovation [43]. However, the micro-mechanism of the Porter hypothesis effect of the EPTL in the specific implementation process has not yet been discussed in depth, nor has there been any analysis of the regulatory behavior of the EPTL, nor has there been any exploration of whether the existing regulatory behavior is conducive to the green technological innovation capability of enterprises. Previous studies believe that compulsory taxation can improve the ESG performance of enterprises and drive the realization of enterprise innovation [44]. However, some studies believe that too stringent environmental regulations will hinder the development of green productivity of enterprises [45][46], reduce the financial performance of enterprises, and squeeze the R&D resources of green technologies [47]. With the maturity of the EPTL system, more and more non-punitive policy tools have begun to play a role, complementing the mandatory tax law, and mobilizing the enthusiasm of enterprises to implement green technology innovation [48][49]. However, the regulatory behavior of the EPTL still plays a major role. Through the study of the existing regulatory behavior, the weak points in the enforcement system of the tax law can be found to better activate the Porter hypothesis effect of the EPTL and promote the development of enterprises' green technology innovation ability.  According to the empirical results of the research model group on the dissimilation effect of green technology innovation regulated by the EPTL, it can be found that some regulatory behaviors are difficult to promote the growth of enterprises' green technology innovation ability. Specifically, the coordination between levies and administrations is only conducive to the realization of end treatment innovation, indicating that there are coordination problems between tax departments and environmental protection departments in the process of tax collection during the implementation of EPTL. For enterprises, meeting the different requirements of the tax department and the environmental protection department in the tax collection process increases the cost of enterprises virtually. Differential tax rate setting is not conducive to the realization of green product innovation, indicating that the existing tax rate level is relatively reasonable, but it is unable to implement a more effective tax rate for product differentiation. Tax information sharing behavior is in a failed state, indicating that it is difficult for enterprises and governments to realize information sharing at the level of environmental protection tax information, thus providing space for the birth of opportunistic behaviors. Definition of the scope of levy and administration behavior is not conducive to the realization of end treatment innovation, which means that the existing environmental protection tax pays insufficient attention to the treatment of wastewater, gas, and slag after production and noise control, and more supervision content is placed on the pre-production and mid-production process.  Tax declaration counseling only affects the end treatment innovation, indicating that the quality of the existing staff is a big problem and can not meet the requirements of environmental regulations on personnel ability. Tax rate level verification only affects the end treatment innovation, which means that the current tax rate of environmental protection tax makes it difficult to stimulate the green technology innovation behavior of most enterprises, the region lacks certain flexibility in the tax rate setting level, and the regional differences are not well reflected in the EPTL. The robustness test results after adding control variables can be found that large and medium-sized enterprises lack green product innovation, and the reason behind this is that the cost of product adjustment for large and medium-sized enterprises exceeds that of small enterprises, technological changes require more resource input, and the implementation of environmental protection tax has squeezed the resources of enterprises, thus making it difficult for green product innovation to reach the expected level. From the perspective of enterprise attributes, the EPTL of foreign-funded enterprises has the most significant regulatory effect, indicating that foreign-funded enterprises have more experience in environmental tax than domestic enterprises, and can quickly adjust corporate behavior according to foreign experience. However, private enterprises do not adapt to the implementation of the EPTL, and the profit-oriented corporate business strategy makes them more inclined to take cunning actions to deal with the environmental tax. From regional perspective, environmental regulation in the central and western regions is not conducive to the realization of enterprises' green technology innovation ability. The reason is that compared with the eastern region, the development of enterprises in the central and western regions depends more on natural resources, and the regulatory behavior has insufficient restraint on regional enterprise behavior, which makes it difficult to stimulate the Porter hypothesis effect.  To achieve environmental protection by using enterprise green technology innovation is the goal of implementing the EPTL, and also the key to the effect of environmental regulation. However, from the empirical test results, the implementation of EPTL at the present stage has produced dissimilation, which seriously affects the realization of green technology innovation ability of enterprises. Although some regulatory behaviors are still playing a role, they have brought serious threats to the transformation and upgrading of enterprise technology structure as a whole, which is not conducive to the sustainable development ability of enterprises, but also makes the EPTL lose its due role. Therefore, to better guide the green technology innovation ability of enterprises and drive the green economy, the regulatory behavior of EPTL needs to be optimized to realize the application of Porter hypothesis effect in the management context of China. |
| REV_  1_4 |  | Literature may be updated by including following studies:  https://doi.org/10.1177/00194662211062428  https://doi.org/10.1002/sd.2797  https://doi.org/10.1016/j.jclepro.2023.139355  https://doi.org/10.1177/0958305X231183921  https://doi.org/10.1108/IJESM-02-2019-0007  https://doi.org/10.1177/2278682117713577  https://doi.org/10.1108/WJSTSD-01-2018-0007  https://doi.org/10.1007/s11356-023-25317-w  https://doi.org/10.1177/0974929217744462  https://doi.org/10.1080/00927678.2019.1639003  https://doi.org/10.1177/2278682116649844 | Thank you for your valuable advice. The author has adjusted the references and partially cited the references provided. |
| REV_  1_5 |  | Language is required through revision | Thank you for your valuable advice. The author has adjusted the language to meet the requirements of language quality. |

# Reviewer#2(REV_2)

| **Co mm ent**  **No.** | **Pa**  **ge**  **No**  **.** | **Reviewer 2: Comments** | **Amendments** |
| --- | --- | --- | --- |
| REV_  2_1 |  | Title  The title "Research on the Path of Green Technology Innovation Driven by the Post-pandemic Era under the Regulation of the Environmental Protection Tax Law" is too broad. The author is advised to reflect the research object and specific content in the title, such as "Research on the Path of Green Technology Innovation Driven by the Environmental Protection Tax Law based on Data of Heavily Polluting Enterprises", to better reflect the actual research content of the article. | Thank you for your valuable comment. The author changed the title to reflect the research content.  **Research on the path of green technology innovation driven by the Environmental Protection Tax Law: Based on data of heavy polluting enterprises** |
| REV_  2_2 |  | Abstract  The abstract still does not accurately summarize the content of the article. The author is advised to re-edit the abstract, highlighting the innovation points of the research, the research methods, and the main findings, while controlling it within 200 words. | Thank you for your valuable comment. The author has rewritten the abstract.  **Abstract:** Environmental Protection Tax Law (EPTL) is a compulsory environmental regulation measure adopted by China to deal with environmental problems. However, with the advancement of implementation, the EPTL produces a dissimilation effect and damages the realization of the Porter hypothesis effect. The study examines the dissimilation effect of green technology innovation regulated by the EPTL using sample data from heavy pollution firms in China. According to the empirical test results: (1) the coordination between levies and administrations, differential tax rate setting, tax information sharing, definition of the scope of levy and administration, tax declaration counseling, and tax rate level verification produce the dissimilation effect; (2) the Porter hypothesis effect of the EPTL is the most significant in medium-sized enterprises and foreign-funded enterprises. By constructing the research model group of dissimilation effect, this study analyzes the application of environmental regulation in China's social and economic background, thus providing a reference for developing of the green economy. |
| REV_  2_3 |  | Theoretical Basis  The analysis of environmental regulation behavior and environmental regulation effect is relatively comprehensive, but too general. The author is advised to simplify and focus the theoretical basis in combination with the research background and data objects of the article. At the same time, the introduction to environmental tax law can be properly simplified. | Thank you for your valuable comment. In the introduction of Section 1, the relevant content is simplified.  1. Introduction  Due to the rapid economic growth, environmental problems have become a key factor restricting the healthy development of China's economy [1]. The relationship between economic growth and environmental protection is not difficult to reconcile, on the contrary, with the help of necessary environmental policy tools can achieve economic and environmental win-win results [2]. Environmental regulation has become an important policy tool to alleviate environmental problems [3]. China also hopes to achieve the common development of the environment and economy with the help of environmental regulation policy tools. On January 1, 2018, the Chinese EPTL was formally implemented, a major event in the course of China's economic and social development, not only marking the formal establishment of China's environmental protection tax system but also demonstrating the Chinese government's attitude and determination to make economic growth and environmental protection a common development objective [4]. The implementation of EPTL in developed countries has a history of more than a hundred years, and rich skills and experience have been accumulated, which has played a positive role in economic growth and environmental protection [5]. However, the normal implementation process of the EPTL in our country has suffered from an abnormal impact and produced the dissimilation effect. In the new economic situation, it is necessary to deeply analyze the dissimilation effect of the EPTL, as well as to propose new corrective and optimization measures to promote the EPTL to play its due value. Thus driving the realization of green technology innovation and effective use of environmental resources [6]. The realization of green technology innovation is of great significance to green economic development and the correction of environmental behavior [7], while the Porter hypothesis effect of EPTL stimulates green technology innovation [8].  The implementation of China’s EPTL has been incubated and explored for more than ten years. In May 2007, China’s State Council issued the Comprehensive Work Program for Energy Conservation and Emission Reduction, which began to explore the issue of environmental taxation. In June 2007, China’s Ministry of Finance (MOF), the General Administration of Taxation (GAT), and the Ministry of Environmental Protection (MEP) set up a Joint Study Group to discuss several issues in the introduction of environmental tax and submitted the draft environmental tax to the State Council at the end of 2014. Subsequently, the Joint Study Group went out for public comment on two more occasions in 2015 and 2016. On December 25, 2016, the Standing Committee of the National People's Congress (NPC) considered and passed the EPTL. On July 21, 2017, China’s MOF, GAT, and MEP jointly promulgated the Notice on Comprehensively Preparing for the Implementation of the EPTL. On December 22, 2017, the State Council issued a Notice on the Attribution of Environmental Protection Tax Revenue. On December 25, 2017, the State Council promulgated the Regulations on the Implementation of the EPTL. On December 27, 2017, the MEP issued the Announcement on the Release of Pollution Coefficients and Material Accounting Methods for Calculating Pollutant Emissions. On January 1, 2018, the EPTL came into effect. On January 7, 2018, the MOF issued the Notice on Matters Related to the Suspension of Sewage Charges and Other Administrative Fees. As an important part of the world, China's environmental behavior will be conducive to the improvement of the global environment. China's EPTL is a mandatory environmental governance strategy led by the government [9], and the key is to achieve a win-win situation between the government's environmental concern and corporate financial performance.  As a typical environmental regulation, the EPTL also has the characteristics of the Porter hypothesis effect [10]. Porter (1991) states that under the right market environment, stringent environmental regulations can incentivize firms to implement in-depth innovation and enhance green technological innovation capabilities, partially or completely offsetting the increase in firms' costs under environmental regulations, thus giving firms a competitive advantage in the marketplace, which is the core idea of the Porter hypothesis [11]. Jaffe and Palmer (1997) based on a large number of enterprise environmental regulation case analyses, and further analysis of the Porter hypothesis, argue that if the enterprise's green technology innovation partially offsets the cost increase under environmental regulation, it is called the Weak Porter hypothesis. If enterprises' green technology innovation completely offsets the cost increase under environmental regulation, it is called the Strong Porter hypothesis [12]. It can be seen that the transition from the Weak Porter hypothesis to the Strong Porter hypothesis is the process of gradually realizing the function of environmental regulation. At the same time, more resources can be allocated to sustainable and environmentally friendly projects, to achieve green development [13]. Therefore, environmental regulations will attract more market participants into the green technology market and achieve green innovation [14].  In environmental economic theory, the Porter hypothesis effect is an effective way to test the effectiveness of the implementation of the EPTL. A few studies show that the Porter hypothesis effect of China’s EPTL has not yet been realized. Based on the sample data of A-share listed companies in Shanghai and Shenzhen from 2011 to 2019, Yuan (2022) utilized the double-difference method and found that the implementation of the EPTL significantly inhibited the green technological innovation capability of enterprises, especially more profoundly and extensively inhibited the green technological innovation capability of state-owned enterprises [15]. The gap between the implementation of environmental laws and regulations and the original intention of legislation makes China's EPTL seriously ineffective [16], which is not conducive to the mitigation of environmental problems, but also hurts the innovation ability of enterprises. In the face of strict EPTL, enterprises will adopt greenwashing behavior to avoid punishment, which is not only detrimental to the shaping of green technology innovation ability but also brings serious harm to the whole society [17].  However, most studies show that the Porter hypothesis effect of China's EPTL has already appeared, but is in an imperfect state. Wen and Zhong (2020) found that the environmental protection tax reform has a significant Porter hypothesis effect on large and medium-sized enterprises, which significantly promotes the growth of green technology innovation in large and medium-sized enterprises, but has no significant impact on small enterprises. Therefore, it is suggested to implement supporting policies to reduce the adverse impact of environmental protection tax on small and micro enterprises [18]. Cui, Lu, and Wang (2021) took China's A-share listed heavy polluting enterprises from 2014 to 2019 as samples and found that the implementation of the Environmental protection tax significantly increased the investment of China's heavy polluting enterprises and enhanced their green technology innovation, and there was still a large space for the promotion of green technology innovation [19]. Leng and Quan (2021) argued that as an environmental regulation, the EPTL has a natural driving effect on green innovation, but at present, the EPTL only has a driving effect on green innovation in the process of production and operation of enterprises, and it can't yet have a driving effect on the green innovation in the result of production and operation [20]. The focus of environmental protection tax also affects the effect of green technology innovation, but the government paying more attention to environmental issues will hurt the finance of enterprises, and thus endanger the investment in technological innovation [21]. Frequent changes in economic policies have inhibited green technology innovation [22], making it difficult to achieve the goal of environmental protection tax. In the study of environmental regulation policy, some scholars believe that relying solely on the mandatory environmental protection tax is not conducive to the green technology innovation ability [23][24][25], which makes it difficult to fully realize the Porte hypothesis effect of EPTL.  Some studies show that China's EPTL not only has the Porter hypothesis effect but also the effect of regional differences on the Porter hypothesis can not be ignored. Hu, Chen, and Zhao (2020) tested the panel data of high-polluting manufacturing enterprises in 30 provincial regions in China from 2008 to 2018 and found that environmental protection tax has a significant promoting effect on green technology innovation of high-polluting enterprises, but the Porter hypothesis effect of environmental protection tax lagging one period is more obvious, and pointed out that regional differences are an important factor affecting the effect of Porter hypothesis of environmental protection tax [26]. Also taking Chinese listed companies from 2010 to 2018 as research objects, Kong et al. (2024) believe that in addition to geographical differences, differences in corporate ownership structure will also have an impact on the effect of environmental protection tax [27].  Existing research shows that China's EPTL has shown the Porter hypothesis effect, which to a certain extent has promoted the green technological innovation of enterprises. However, with the advancement of the process, the promoting effect of green technology innovation is declining. Existing studies have rarely mentioned the alienating effect of environmental protection tax in China, nor have they discussed the internal mechanism in-depth, nor have they conducted a comprehensive review of the promoting effect of environmental protection tax on the technological innovation ability of enterprises. As a compulsory environmental regulation, the environmental regulation behavior and the environmental regulation effect of environmental protection tax have not been fully discussed. Therefore, to implement the EPTL and get rid of the framework of dissimilation effect as soon as possible, it is necessary to deeply analyze the dissimilation mechanism of the environmental protection tax collection and management, and propose improvement plans to improve the quality of the environmental protection tax collection and management, to maximize the green technological innovation and corporate competitive advantage of enterprises.  The innovations of this paper can be summarized as follows: First, the impact of environmental regulations on green technology innovation is explored, revealing the role of government controls on firms' technological development. Second, the green technology innovation driving path based on China's environmental protection tax is explored through empirical tests. Thirdly, the paper analyzes the role of environmental regulation in China's social and economic background under the framework of the dissimilation effect and further verifies the role of the Porter hypothesis in China's environmental background by constructing a research model set of dissimilation effects. |
| REV_  2_4 |  | Research Design  (1) In terms of sample selection, the author's approach of having executives of heavily polluting enterprises fill out questionnaires is reasonable, but the description of sample characteristics is too simple. The author is advised to supplement more descriptive statistical data of the samples. | Thank you for your valuable comment. The data of sample descriptive statistical analysis were supplemented. The descriptive statistical characteristics of the sample are shown in Table 3. Table 3 Descriptive statistical characteristics of samples Variable name \| Maximum value \| Minimum value \| Mode \| Mean value \| Variance; Coordination between levies and administrations ( \| 7 \| 1 \| 2 \| 2.19 \| 0.09; Emissions monitoring science ( \| 7 \| 1 \| 4 \| 3.87 \| 0.16; Differential tax rate setting ( \| 7 \| 1 \| 3 \| 3.11 \| 0.12; Tax information sharing ( \| 7 \| 1 \| 2 \| 2.29 \| 0.10; Definition of the scope of levy and administration ( \| 7 \| 1 \| 3 \| 2.98 \| 0.13; Tax declaration counseling ( \| 7 \| 1 \| 2 \| 1.99 \| 0.07; Accountability mechanism design ( \| 7 \| 1 \| 3 \| 3.08 \| 0.12; Implementation of preferential policies ( \| 7 \| 1 \| 4 \| 3.91 \| 0.15; Tax rate level verification ( \| 7 \| 1 \| 2 \| 2.22 \| 0.08; Green process innovation ( \| 7 \| 1 \| 3 \| 2.88 \| 0.13; Green product innovation ( \| 7 \| 1 \| 3 \| 3.03 \| 0.16; End treatment innovation ( \| 7 \| 1 \| 3 \| 3.27 \| 0.10; Green technology innovation ( \| 7 \| 1 \| 3 \| 2.91 \| 0.12 |
| REV_  2_5 |  | (2) In terms of variable setting, the control variables only consider the size and attributes of the enterprise. The author is advised to further expand the scope of control variables, such as regional differences and industry differences, to improve the accuracy and credibility of the research. | Thank you for your valuable comments. In order to further improve the accuracy and credibility of the study, regional difference was added as a control variable in the robustness test, and the research model was re-tested. Table 4 Test results of the research model \| Green process innovation ($Gcra$) \| Green product innovation ($Gcom$) \| End treatment innovation ($Gend$) \| Green technology innovation ($Gtec$); Coordination between levies and administrations ($xt)$ \| 0.05(0.0632) \| 0.03(0.0702) \| 0.12**(0.0072) \| 0.07(0.0611); Emissions monitoring science ($jc)$ \| 0.12**(0.0063) \| 0.13***(0.0005) \| 0.15***(0.0008) \| 0.14***(0.0003); Differential tax rate setting $(cy)$ \| 0.09*(0.0315) \| 0.07(0.0858) \| 0.11**(0.0076) \| 0.10*(0.0332); Tax information sharing $(gx)$ \| 0.02(0.1121) \| 0.01(0.0871) \| 0.03(0.0772) \| 0.00(0.0518); Definition of the scope of levy and administration ($fw)$ \| 0.08*(0.0405) \| 0.10**(0.0059) \| 0.07(0.0606) \| 0.09*(0.0323); Tax declaration counseling $(fd)$ \| 0.07(0.0922) \| 0.05(0.0688) \| 0.09*(0.0303) \| 0.03(0.0627); Accountability mechanism design ($wz)$ \| 0.11**(0.0059) \| 0.09*(0.0401) \| 0.13***(0.0005) \| 0.10**(0.0027); Implementation of preferential policies ($yh)$ \| 0.13***(0.0006) \| 0.08*(0.0328) \| 0.15***(0.0008) \| 0.12***(0.0006); Tax rate level verification $(sl)$ \| 0.02(0.1031) \| 0.03(0.0962) \| 0.10*(0.0356) \| 0.06(0.0708); Medium-sized enterprise $(me)$ \| 0.12**(0.0059) \| 0.07(0.0602) \| 0.14***(0.0002) \| 0.13***(0.0004); Large-sized enterprise $(be)$ \| 0.10*(0.0202) \| 0.05(0.0662) \| 0.11***(0.0007) \| 0.09*(0.0215); $R^{2}$ \| 0.69 \| 0.70 \| 0.62 \| 0.66; $\Delta R^{2}$ \| 0.01 \| 0.02 \| 0.02 \| 0.01; adjusted$R^{2}$ \| 0.70 \| 0.72 \| 0.64 \| 0.67; adjusted $F$Value \| 108.36 \| 88.19 \| 79.26 \| 138.76; $P$Value \| **(0.0033) \| ***(0.0004) \| **(0.0052) \| ***(0.0005) Notes: *P<0.05; **P<0.01; ***P<0.001; N=428; The values in parentheses are P value. Based on the initial test of the research model, the enterprise attribute type and area attribute type are continued to be added as control variables, and the robustness test of the research model is carried out, the test results are shown in Table 5. A comparison of Table 5 with Table 4 reveals that the model test is robust. Table 5 Test results of the research model (robustness test) \| Green process innovation ($Gcra$) \| Green product innovation ($Gcom$) \| End treatment innovation ($Gend$) \| Green technology innovation ($Gtec$); Coordination between levies and administrations ($xt)$ \| 0.07(0.1032) \| 0.05(0.1164) \| 0.12**(0.0062) \| 0.08(0.0645); Emissions monitoring science ($jc)$ \| 0.12**(0.0084) \| 0.13***(0.0007) \| 0.12**(0.0067) \| 0.11**(0.0052); Differential tax rate setting $(cy)$ \| 0.10*(0.0427) \| 0.03(0.0976) \| 0.13**(0.0084) \| 0.10*(0.0382); Tax information sharing $(gx)$ \| 0.05(0.1028) \| 0.01(0.0981) \| 0.05(0.0655) \| 0.03(0.0852); Definition of the scope of levy and administration ($fw)$ \| 0.11*(0.0452) \| 0.13**(0.0086) \| 0.07(0.0583) \| 0.13*(0.0355); Tax declaration counseling $(fd)$ \| 0.07(0.1076) \| 0.05(0.0927) \| 0.11*(0.0337) \| 0.05(0.0876); Accountability mechanism design ($wz)$ \| 0.11**(0.0052) \| 0.12*(0.0257) \| 0.13**(0.0076) \| 0.11*(0.0411); Implementation of preferential policies ($yh)$ \| 0.10**(0.0064) \| 0.11*(0.0322) \| 0.12**(0.0083) \| 0.14***(0.0007); Tax rate level verification $(sl)$ \| 0.02(0.0623) \| 0.05(0.0877) \| 0.10*(0.0201) \| 0.06(0.0768); Medium-sized enterprise $(me)$ \| 0.15***(0.0004) \| 0.07(0.0966) \| 0.13**(0.0064) \| 0.15***(0.0006); Large-sized enterprise $(be)$ \| 0.14**(0.0065) \| 0.02(0.0662) \| 0.12**(0.0072) \| 0.13*(0.0352); Private enterprise $(pe)$ \| -0.13***(0.0005) \| -0.11**(0.0056) \| -0.12**(0.0084) \| -0.14***(0.0006); Foreign-funded enterprise $(fe)$ \| 0.10*(0.0266) \| 0.11*(0.0314) \| 0.12**(0.0056) \| 0.16***(0.0004); Central China ($mi)$ \| -0.08(0.0866) \| -0.10*(0.0288) \| -0.02(0.0705) \| -0.12**(0.0064); Western China ($we)$ \| -0.11**(0.0047) \| -0.02(0.1035) \| -0.14***(0.0005) \| -0.02(0.0688); $R^{2}$ \| 0.72 \| 0.68 \| 0.66 \| 0.67; $\Delta R^{2}$ \| 0.05 \| 0.02 \| 0.02 \| 0.04; adjusted$R^{2}$ \| 0.77 \| 0.70 \| 0.68 \| 0.71; adjusted $F$Value \| 123.29 \| 92.78 \| 100.26 \| 87.19; $P$Value \| **(0.0067) \| **(0.0026) \| ***(0.0008) \| ***(0.0005) Notes: *P<0.05; **P<0.01; ***P<0.001; N=428; The values in parentheses are P value. |
| REV_  2_6 |  | (3) In terms of model construction, the author considers the two levels of environmental regulation behavior and effect, and the research ideas are clear. However, the details of specific variable setting and model formula are not sufficiently elaborated. The author is advised to further explain and improve this part. | Thank you for your valuable comments. The details of the model formula and the attributes of the variables have been explained in detail in Table 1 Variable characteristics. Table 1 Variable characteristics Variable name \| Variable symbol \| Variable coefficient \| Variable connotation; **Main independent variable** \| \| \|; Coordination between levies and administrations \|  \|  \| Coordination between tax levies and environmental authorities in the tax collection process.; Emissions monitoring science \|  \|  \| The monitoring of pollution emissions from enterprises by the environmental protection authorities is scientific and accurate.; Differential tax rate setting \|  \|  \| Setting different tax rates or amounts for different industries and different pollutant emissions.; Tax information sharing \|  \|  \| Information and data on the collection and payment of environmental protection taxes can be shared by both the collector and the payer.; Definition of the scope of levy and administration \|  \|  \| The subject or scope of the environmental protection tax includes all pollution emissions.; Tax declaration counseling \|  \|  \| Tax authorities guide enterprises on tax accounting, declaration, reduction, and exemption.; Accountability mechanism design \|  \|  \| Accountability mechanisms have been put in place and have created good norms for the conduct of levies.; Implementation of preferential policies \|  \|  \| The preferential policies of environmental protection tax reduction and exemption can be open, just, and fair.; Tax rate level verification \|  \|  \| The setting of regional tax rates can promote energy conservation and emission reduction for most enterprises.; **Controlled variable** \| \| \|; Small enterprise \| \| \| Basic variable, do not participate in the model testing.; Medium-sized enterprise \|  \|  \| Binary variable, the value of 1 indicates that the enterprise is a medium-sized heavy polluting enterprise.; Large-sized enterprise \|  \|  \| Binary variable, the value of 1 indicates that the enterprise is a large-sized heavy polluting enterprise.; State-owned enterprise \| \| \| Basic variable, do not participate in the model testing.; Private enterprise \|  \|  \| Binary variable, the value of 1 indicates that the enterprise is a private heavy polluting enterprise.; Foreign-funded enterprise \|  \|  \| Binary variable, the value of 1 indicates that the enterprise is a foreign-funded heavy polluting enterprise.; Eastern China \| \| \| Basic variable, do not participate in the model testing.; Central China \| *mi* \|  \| Binary variable, the value of 1 indicates that the enterprise is located in Central China.; Western China \| *we* \|  \| Binary variable, the value of 1 indicates that the enterprise is located in Western China.; **Explained variable** \| \| \|; Green process innovation \|  \| \| Environmental innovations in process design, production flow, and supply chain of heavily polluting enterprises.; Green product innovation \|  \| \| Environmental protection innovation in product transportation, consumption, application, and recycling of heavy polluting enterprises; End treatment innovation \|  \| \| Environmental innovations in wastewater, gas, sludge treatment, and noise control of heavy polluting enterprises.; Green technology innovation \|  \| \| Environmental protection innovation of heavy polluting enterprises in the whole operation process or market competition. |
| REV_  2_7 |  | Empirical Results  (1) The results reporting is not rigorous enough. The author directly lists the coefficient values of the model regression results, but lacks necessary statistical explanations of these values, such as the significance level of the coefficients. | Thanks for your valuable advice. The significance levels of the coefficients have been described and illustrated using p-values. If you use words to elaborate, it will make the paper appear bloated. |
| REV_  2_8 |  | (2) The results analysis lacks depth. The author only makes a simple description of the regression results, without in-depth discussion of the mechanism of action of different variables and their economic significance. | Thanks for your valuable advice. The author completed the discussion of the result analysis by supplementing the discussion part.  *3.3* Discussion  The study empirically tests the effects of the Porter hypothesis of the EPTL by constructing a research model set of the dissimilation effects of green technological innovations regulated by the EPTL and utilizing sample data from heavy polluting enterprises in China. According to the results of the model test, it can be seen that with the implementation of China's EPTL, the promotion effect of environmental regulation on green technological innovation has been alienated, and the effect of Porter hypothesis has failed to be fully realized, and the realization of the green technological innovation ability of heavy polluting enterprises has been impeded by the existing regulatory behavior.  The environmental regulation effect of the EPTL has been proved [42], and the effective implementation of the tax law can urge enterprises to take positive measures to deal with environmental punishment, to increase their investment in green technology innovation [43]. However, the micro-mechanism of the Porter hypothesis effect of the EPTL in the specific implementation process has not yet been discussed in depth, nor has there been any analysis of the regulatory behavior of the EPTL, nor has there been any exploration of whether the existing regulatory behavior is conducive to the green technological innovation capability of enterprises. Previous studies believe that compulsory taxation can improve the ESG performance of enterprises and drive the realization of enterprise innovation [44]. However, some studies believe that too stringent environmental regulations will hinder the development of green productivity of enterprises [45][46], reduce the financial performance of enterprises, and squeeze the R&D resources of green technologies [47]. With the maturity of the EPTL system, more and more non-punitive policy tools have begun to play a role, complementing the mandatory tax law, and mobilizing the enthusiasm of enterprises to implement green technology innovation [48][49]. However, the regulatory behavior of the EPTL still plays a major role. Through the study of the existing regulatory behavior, the weak points in the enforcement system of the tax law can be found to better activate the Porter hypothesis effect of the EPTL and promote the development of enterprises' green technology innovation ability.  According to the empirical results of the research model group on the dissimilation effect of green technology innovation regulated by the EPTL, it can be found that some regulatory behaviors are difficult to promote the growth of enterprises' green technology innovation ability. Specifically, the coordination between levies and administrations is only conducive to the realization of end treatment innovation, indicating that there are coordination problems between tax departments and environmental protection departments in the process of tax collection during the implementation of EPTL. For enterprises, meeting the different requirements of the tax department and the environmental protection department in the tax collection process increases the cost of enterprises virtually. Differential tax rate setting is not conducive to the realization of green product innovation, indicating that the existing tax rate level is relatively reasonable, but it is unable to implement a more effective tax rate for product differentiation. Tax information sharing behavior is in a failed state, indicating that it is difficult for enterprises and governments to realize information sharing at the level of environmental protection tax information, thus providing space for the birth of opportunistic behaviors. Definition of the scope of levy and administration behavior is not conducive to the realization of end treatment innovation, which means that the existing environmental protection tax pays insufficient attention to the treatment of wastewater, gas, and slag after production and noise control, and more supervision content is placed on the pre-production and mid-production process.  Tax declaration counseling only affects the end treatment innovation, indicating that the quality of the existing staff is a big problem and can not meet the requirements of environmental regulations on personnel ability. Tax rate level verification only affects the end treatment innovation, which means that the current tax rate of environmental protection tax makes it difficult to stimulate the green technology innovation behavior of most enterprises, the region lacks certain flexibility in the tax rate setting level, and the regional differences are not well reflected in the EPTL. The robustness test results after adding control variables can be found that large and medium-sized enterprises lack green product innovation, and the reason behind this is that the cost of product adjustment for large and medium-sized enterprises exceeds that of small enterprises, technological changes require more resource input, and the implementation of environmental protection tax has squeezed the resources of enterprises, thus making it difficult for green product innovation to reach the expected level. From the perspective of enterprise attributes, the EPTL of foreign-funded enterprises has the most significant regulatory effect, indicating that foreign-funded enterprises have more experience in environmental tax than domestic enterprises, and can quickly adjust corporate behavior according to foreign experience. However, private enterprises do not adapt to the implementation of the EPTL, and the profit-oriented corporate business strategy makes them more inclined to take cunning actions to deal with the environmental tax. From regional perspective, environmental regulation in the central and western regions is not conducive to the realization of enterprises' green technology innovation ability. The reason is that compared with the eastern region, the development of enterprises in the central and western regions depends more on natural resources, and the regulatory behavior has insufficient restraint on regional enterprise behavior, which makes it difficult to stimulate the Porter hypothesis effect.  To achieve environmental protection by using enterprise green technology innovation is the goal of implementing the EPTL, and also the key to the effect of environmental regulation. However, from the empirical test results, the implementation of EPTL at the present stage has produced dissimilation, which seriously affects the realization of green technology innovation ability of enterprises. Although some regulatory behaviors are still playing a role, they have brought serious threats to the transformation and upgrading of enterprise technology structure as a whole, which is not conducive to the sustainable development ability of enterprises, but also makes the EPTL lose its due role. Therefore, to better guide the green technology innovation ability of enterprises and drive the green economy, the regulatory behavior of EPTL needs to be optimized to realize the application of Porter hypothesis effect in the management context of China. |
| REV_  2_9 |  | (3) The setting of the robustness test is relatively reasonable, but the interpretation of the results is also insufficient. | Thanks for your valuable advice. The results are explained in the discussion section.  *3.3* Discussion  The study empirically tests the effects of the Porter hypothesis of the EPTL by constructing a research model set of the dissimilation effects of green technological innovations regulated by the EPTL and utilizing sample data from heavy polluting enterprises in China. According to the results of the model test, it can be seen that with the implementation of China's EPTL, the promotion effect of environmental regulation on green technological innovation has been alienated, and the effect of Porter hypothesis has failed to be fully realized, and the realization of the green technological innovation ability of heavy polluting enterprises has been impeded by the existing regulatory behavior.  The environmental regulation effect of the EPTL has been proved [42], and the effective implementation of the tax law can urge enterprises to take positive measures to deal with environmental punishment, to increase their investment in green technology innovation [43]. However, the micro-mechanism of the Porter hypothesis effect of the EPTL in the specific implementation process has not yet been discussed in depth, nor has there been any analysis of the regulatory behavior of the EPTL, nor has there been any exploration of whether the existing regulatory behavior is conducive to the green technological innovation capability of enterprises. Previous studies believe that compulsory taxation can improve the ESG performance of enterprises and drive the realization of enterprise innovation [44]. However, some studies believe that too stringent environmental regulations will hinder the development of green productivity of enterprises [45][46], reduce the financial performance of enterprises, and squeeze the R&D resources of green technologies [47]. With the maturity of the EPTL system, more and more non-punitive policy tools have begun to play a role, complementing the mandatory tax law, and mobilizing the enthusiasm of enterprises to implement green technology innovation [48][49]. However, the regulatory behavior of the EPTL still plays a major role. Through the study of the existing regulatory behavior, the weak points in the enforcement system of the tax law can be found to better activate the Porter hypothesis effect of the EPTL and promote the development of enterprises' green technology innovation ability.  According to the empirical results of the research model group on the dissimilation effect of green technology innovation regulated by the EPTL, it can be found that some regulatory behaviors are difficult to promote the growth of enterprises' green technology innovation ability. Specifically, the coordination between levies and administrations is only conducive to the realization of end treatment innovation, indicating that there are coordination problems between tax departments and environmental protection departments in the process of tax collection during the implementation of EPTL. For enterprises, meeting the different requirements of the tax department and the environmental protection department in the tax collection process increases the cost of enterprises virtually. Differential tax rate setting is not conducive to the realization of green product innovation, indicating that the existing tax rate level is relatively reasonable, but it is unable to implement a more effective tax rate for product differentiation. Tax information sharing behavior is in a failed state, indicating that it is difficult for enterprises and governments to realize information sharing at the level of environmental protection tax information, thus providing space for the birth of opportunistic behaviors. Definition of the scope of levy and administration behavior is not conducive to the realization of end treatment innovation, which means that the existing environmental protection tax pays insufficient attention to the treatment of wastewater, gas, and slag after production and noise control, and more supervision content is placed on the pre-production and mid-production process.  Tax declaration counseling only affects the end treatment innovation, indicating that the quality of the existing staff is a big problem and can not meet the requirements of environmental regulations on personnel ability. Tax rate level verification only affects the end treatment innovation, which means that the current tax rate of environmental protection tax makes it difficult to stimulate the green technology innovation behavior of most enterprises, the region lacks certain flexibility in the tax rate setting level, and the regional differences are not well reflected in the EPTL. The robustness test results after adding control variables can be found that large and medium-sized enterprises lack green product innovation, and the reason behind this is that the cost of product adjustment for large and medium-sized enterprises exceeds that of small enterprises, technological changes require more resource input, and the implementation of environmental protection tax has squeezed the resources of enterprises, thus making it difficult for green product innovation to reach the expected level. From the perspective of enterprise attributes, the EPTL of foreign-funded enterprises has the most significant regulatory effect, indicating that foreign-funded enterprises have more experience in environmental tax than domestic enterprises, and can quickly adjust corporate behavior according to foreign experience. However, private enterprises do not adapt to the implementation of the EPTL, and the profit-oriented corporate business strategy makes them more inclined to take cunning actions to deal with the environmental tax. From regional perspective, environmental regulation in the central and western regions is not conducive to the realization of enterprises' green technology innovation ability. The reason is that compared with the eastern region, the development of enterprises in the central and western regions depends more on natural resources, and the regulatory behavior has insufficient restraint on regional enterprise behavior, which makes it difficult to stimulate the Porter hypothesis effect.  To achieve environmental protection by using enterprise green technology innovation is the goal of implementing the EPTL, and also the key to the effect of environmental regulation. However, from the empirical test results, the implementation of EPTL at the present stage has produced dissimilation, which seriously affects the realization of green technology innovation ability of enterprises. Although some regulatory behaviors are still playing a role, they have brought serious threats to the transformation and upgrading of enterprise technology structure as a whole, which is not conducive to the sustainable development ability of enterprises, but also makes the EPTL lose its due role. Therefore, to better guide the green technology innovation ability of enterprises and drive the green economy, the regulatory behavior of EPTL needs to be optimized to realize the application of Porter hypothesis effect in the management context of China. |
| REV_  2_10 |  | Conclusions and Policy Recommendations  (1) The conclusion part summarizes the empirical findings comprehensively, but the author is advised to grade the importance of each conclusion point, and better highlight the main findings. | Thanks for your valuable advice. The authors reframe the conclusion, highlighting the main findings.  4.1 Conclusions  Through the comprehensive judgment of the test results, the following conclusions can be drawn. First, the environmental regulatory acts of the EPTL, such as the coordination of collection and management departments, the sharing of tax collection information, the counseling on tax declaration, and the approval of tax rate levels, have not produced a substantial effect on the promotion of enterprises' green technological innovation, while the rest of the regulatory acts have facilitated the growth of the enterprises' green technological innovation capacity to varying degrees. Second, the Porter hypothesis effect of the EPTL is most pronounced in medium-sized enterprises, weakest in large-sized enterprises, and second weakest in small enterprises. Third, the Porter hypothesis effect of the EPTL is the most obvious in foreign-funded enterprises, followed by state-owned enterprises, and the weakest in private enterprises.  In conclusion, with the implementation of the EPTL, regulatory behavior difficult to ensure the effectiveness of the EPTL. The main performance is Porter hypothesis effect has been significantly weakened, the driving force for enterprises' green technology innovation is decreased, and most regulatory behaviors hinder the occurrence of enterprises' green technology innovation. Therefore, this unfavorable situation should be reversed as soon as possible, the Porter hypothesis effect should be restored, and the environmental regulatory function of the EPTL should be fully realized, to vigorously enhance the green technological innovation capability of enterprises.  By constructing the dissimilarity effect research model group to empirically test the green technology innovation driving path of China's environmental protection tax, this study expands the application of the Porter hypothesis effect in China's management context, further reveals the micro-mechanism of the environmental regulatory effect of the EPTL, and provides theoretical references and practical bases for the realization of the co-development of the economy and the environment for the rest of the world countries and regions. |
| REV_  2_11 |  | (2) The policy recommendations part puts forward some reasonable insights, but overall the policy recommendations are still thin, lacking necessary details and quantitative analysis support. | Thanks for your valuable advice. The author made some changes to the countermeasure part and rewrote it according to the opinions of other reviewers.  4.2 Policy implications  According to the test results, combined with the investigation on the implementation of China's EPTL, it can be seen that to more effectively drive the growth of green technological innovation in enterprises, the reform and improvement of the EPTL should be focused on promoting in the following directions. First, strengthen the contact and communication between the tax department and the environmental protection department at the senior management. According to the empirical test results, the coordination between levies and administrations has not achieved the drive of green process innovation, green product innovation, and green technology innovation. Therefore, it is necessary to break the business gap between the tax department and the environmental protection department, improve the coordination in the operation process, and ensure the effect of the implementation of the EPTL. Second, local government departments need to vigorously promote the construction of information sharing platforms for environmental protection tax. According to the empirical test results, the regulatory behavior of tax information sharing does not play a driving role in the green technology innovation ability of enterprises. Local governments need to personally guide environmental protection agencies, tax agencies, and tax-paying enterprises to participate together, to vigorously promote the process of information sharing platform construction. Third, the tax authorities need to increase the business training of enterprise taxpayers. According to the empirical test results, the regulation behavior of tax declaration counseling has a serious alienation effect, which only plays a role in the end treatment innovation. Therefore, the tax authorities need to send experienced tax personnel to participate in the training work and change the training method to open the training situation as soon as possible. Finally, flexibly adjust the tax rate level of regional environmental protection tax. According to the empirical test results, the regulatory behavior of tax rate level verification is conducive to end-treatment innovation, but not conducive to green process innovation, green product innovation, and green technology innovation. Therefore, each region should establish the most effective tax rate according to their economy, society, and environmental protection, to promote the dual development of environmental protection and economic growth. |
| REV_  2_12 |  | (3) Although the innovation points are summarized, the discussion is not concise enough. The author is advised to refine the innovation points in 1-2 core sentences. | Thank you for your valuable comments. The author re-summarizes the innovative points to make it more concise.  4.1 Conclusions  By constructing the dissimilarity effect research model group to empirically test the green technology innovation driving path of China's environmental protection tax, this study expands the application of the Porter hypothesis effect in China's management context, further reveals the micro-mechanism of the environmental regulatory effect of the EPTL, and provides theoretical references and practical bases for the realization of the co-development of the economy and the environment for the rest of the world countries and regions. |
| REV_  2_13 |  | Language and Format  (1) There are some problems with the format of data expression and formula numbering, and the author is advised to unify the format. | Thank you for your valuable comments. The authors unify the format of data expression and formula number. |
| REV_  2_14 |  | (2) The normative of the cited references needs to be further improved. | Thank you for your valuable comments. According to the requirements of the journal, the author has perfected the standard of the cited references. |
